# Classes of tree-based networks

**DOI:** 10.1186/s42492-020-00043-z

**Published:** 2020-05-15

**Authors:** Mareike Fischer, Michelle Galla, Lina Herbst, Yangjing Long, Kristina Wicke

**Affiliations:** 1 Institute of Mathematics and Computer Science, University of Greifswald, Walther-Rathenau-Straße 47, 17489 Greifswald, Germany; 2 School of Mathematics and Statistics, Central China Normal University, 800 Dongchuan Road, Shanghai, 200240 China

**Keywords:** Phylogenetic tree, Phylogenetic network, Tree-based network, Edge-based network, Chordal network, Hamilton connected, Hamiltonian path, Generalized series-parallel graphs, Series-parallel graphs

## Abstract

Recently, so-called tree-based phylogenetic networks have attracted considerable attention. These networks can be constructed from a phylogenetic tree, called the base tree, by adding additional edges. The primary aim of this study is to provide sufficient criteria for tree-basedness by reducing phylogenetic networks to related graph structures. Even though it is generally known that determining whether a network is tree-based is an NP-complete problem, one of these criteria, namely edge-basedness, can be verified in linear time. Surprisingly, the class of edge-based networks is closely related to a well-known family of graphs, namely, the class of generalized series-parallel graphs, and we explore this relationship in full detail. Additionally, we introduce further classes of tree-based networks and analyze their relationships.

## Introduction

Phylogenetic networks are of considerable interest, as they allow the representation of non-treelike evolutionary events, such as hybridization and horizontal gene transfer.

Various classes of phylogenetic networks have been introduced and studied. One of them is the class of so-called tree-based networks. Roughly, a phylogenetic network is tree-based if it can be obtained from a phylogenetic tree by adding additional edges.

[1] first introduced this concept for binary rooted phylogenetic networks, and more recently, [2] extended it to binary unrooted networks, [3] to non-binary rooted networks, and [4, 5] to non-binary unrooted networks.

In the present study, we focus on unrooted networks and consider both the binary and non-binary cases.

We first introduce three procedures that reduce a phylogenetic network to related graphs. This leads to sufficient criteria ensuring that a phylogenetic network is tree-based (whether it is binary or not). Some of these criteria are based on classical graph theory, particularly on the theory of Hamiltonian paths, cycles, and graphs. Another sufficient criterion for tree-basedness is a property to which we refer as edge-basedness. This criterion is again related to classical graph theory, namely, to generalized series-parallel graphs (GSP graphs). We will introduce this concept in full detail, highlight the relationship between edge-based graphs and GSP graphs and analyze its implications. In particular, we remark that edge-basedness can be tested in linear time because GSP graphs can be recognized in linear time. This is also of practical relevance, as in general, the problem of determining whether a network is tree-based is NP-complete [2].

The remainder of this paper is organized as follows. In Section Methods, we introduce some basic phylogenetic and graph-theoretical concepts and terminology. We then introduce three procedures: leaf cutting, shrinking, and connecting. These reduce a phylogenetic network to related graphs. This leads to sufficient criteria for tree-basedness (e.g., edge-basedness) and some classes of phylogenetic networks that are necessarily tree-based. After summarizing the relationships between these classes, we conclude the paper in Section Discussion and Conclusion, where we discuss our results and indicate possible directions of future research.

## Methods

We use mathematical proofs based on the definitions and methods presented in this section.

### Phylogenetic and basic graph-theoretical concepts

Throughout this paper, *G* = (*V*(*G*), *E*(*G*)) (or *G* = (*V*, *E*) for brevity) will denote a graph with vertex set *V*(*G*) and edge set *E*(*G*). We note that in this study, graphs may contain parallel edges and loops. If we require graphs without parallel edges and/or loops, we will specifically use the term simple graphs, and when parallel edges are allowed but loops are not, we will use the term loopless graphs. Furthermore, we will use the notation *N*_*G*_(*v*) (or *N*(*v*) for brevity if there is no ambiguity) to denote the neighborhood of a vertex *v* in *G*, that is, the set of vertices adjacent to *v* in *G*. We note that if *G* is a simple graph without parallel edges and loops, we have ∣*N*_*G*_(*v*) ∣  = deg(*v*).

Let now *X* denote a finite set (e.g., of taxa or species) with |*X*| ≥ 1. An unrooted phylogenetic network *N*^*u*^ (on *X*) is a connected simple graph *G* = (*V*, *E*) with *X* ⊆ *V* and no vertices of degree 2, where the set of degree-1 vertices (referred to as the leaves or taxa of the network) is bijectively labeled by *X*. Such an unrooted network is called unrooted binary if every inner vertex *u* ∈ *V* ∖ *X* has degree 3. It is called a phylogenetic tree if the underlying graph structure is a tree. In the following, we denote by $\dot{E}$ the set of inner edges of *N*^*u*^, that is, those edges that are not incident to a leaf. A phylogenetic network *N*^*u*^ = (*V*, *E*) on *X* is called tree-based if there is a spanning tree *T* = (*V*, *E*′) in *N*^*u*^ (with *E*′ ⊆ *E*) whose leaf set is equal to *X*. This spanning tree is then called a support tree for *N*^*u*^. Moreover, the tree *T*′ that can be obtained from *T* by suppressing potential degree-2 vertices is called a base tree for *N*^*u*^. We note that the existence of a support tree *T* for *N*^*u*^ implies the existence of a base tree *T*′ for *N*^*u*^.

In the analysis of networks, or more generally, connected graphs, it is often useful to decompose them into simpler parts, which can then be analyzed individually. Therefore, let *G* = (*V*, *E*) be a connected graph. A cut edge, or bridge, of *G* is an edge *e* whose removal disconnects the graph. Similarly, a vertex *v* is a cut vertex (sometimes also called an articulation) if deleting *v* and all its incident edges disconnects the graph. Moreover, a set $\mathcal{C}$ of vertices whose removal disconnects the graph is called a separating set or vertex cut.

If after the removal of a cut edge, one of the induced connected components of the resulting graph is a single vertex, the corresponding cut edge is called trivial. We call *N*^*u*^ a simple network if all of its cut edges are trivial.

**Fig. 1:**
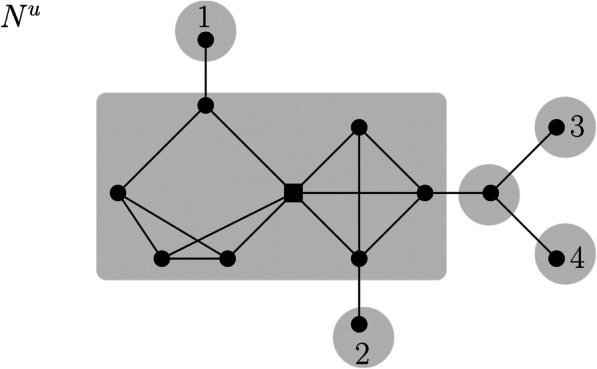
Unrooted non-binary phylogenetic network N^u^ on leaves 1, 2, 3, and 4. The gray areas correspond to the blobs of N^u^. Notice that the biggest blob contains a cut vertex (depicted as a square vertex). Moreover, notice that N^u^ can be considered as a tree with blobs as vertices, as the cut edges and blobs of N^u^ induce a “tree structure”

A blob in a connected graph (and more specifically, in a network) is a maximal connected subgraph that has no cut edge. Note, however, that a blob may contain cut vertices. An example of such a blob can be seen in Fig. 1. Moreover, we note that we consider a network to be a “tree” with blobs as vertices [6]. In contrast, a block in a connected graph *G* is a maximal biconnected subgraph of *G*, that is, a maximal induced subgraph that remains connected if any of its vertices is removed. In particular, a block does not contain cut vertices.

Following [5], we call a graph *G* (or a network *N*^*u*^) proper if the removal of any cut edge or cut vertex in the graph (or the network) leads to connected components, each containing at least one leaf.

Finally, two important operations on graphs that will be used in the following are edge subdivision and vertex suppression. Let now *G* be a graph with some edge *e* = {*u*, *v*}. Then, we say that we subdivide *e* by deleting *e*, adding a new vertex *w*, and adding the edges {*u*, *w*} and {*w*, *v*}. The new degree-2 vertex *w* is sometimes also called an attachment point. We note that we also often refer to the vertex adjacent to a vertex *x* of degree 1 (i.e., adjacent to a leaf *x*) as the attachment point of *x*, even if it is a vertex of degree higher than 2. Conversely, given a degree-2 vertex *w* with adjacent vertices *u* and *v*, suppressing *w* implies deleting *w* and its two incident edges {*u*, *w*} and {*w*, *v*}, and adding a new edge {*u*, *v*}.

### Further graph-theoretical concepts

Before we can introduce three procedures for reducing a phylogenetic network to related graphs, we recall some basic concepts from classical graph theory. Most importantly, we recall the notion of Hamiltonian paths and Hamiltonian cycles.

A Hamiltonian path in a graph is a path that visits each vertex exactly once. If this path is a cycle, we call the path a Hamiltonian cycle. Moreover, a graph that contains a Hamiltonian cycle is called a Hamiltonian graph. A graph is called Hamilton connected if for every two vertices *u*, *v*, there is a Hamiltonian path from *u* to *v*. In particular, we note that every Hamilton connected graph is Hamiltonian because the strong property of Hamilton connectedness also holds for adjacent vertices, so that the edge *e* = {*u*, *v*} together with the Hamiltonian path from *u* to *v* forms a Hamiltonian cycle. As has been noted by [2], there is a strong connection between Hamiltonian paths and tree-basedness of phylogenetic networks. However, before we can elaborate on this in more detail, we should introduce a few more concepts.

We first recall that the toughness *t*(*G*) of a graph *G* (or, analogously, of a phylogenetic network *N*^*u*^) is defined as

$$ t(G)=\underset{\mathcal{C}}{\min}\frac{\left|\mathcal{C}\right|}{c\left(G-\mathcal{C}\right)} $$

where the minimum is taken over all separating sets $\mathcal{C}$ of *G*, *G* − $\mathcal{C}$ denotes the (disconnected) graph that is obtained by deleting all vertices of $\mathcal{C}$ from *G* and all edges incident to $\mathcal{C},$ and $c\left(G-\mathcal{C}\right)$ denotes the number of connected components in $G-\mathcal{C}$. The concept of toughness plays an important role in the study of Hamiltonian graphs [7, 8], and thus, as we will show, for tree-basedness of a network as well.

Subsequently, we will consider chordal graphs. We recall that a graph is called chordal if each cycle of length 4 or more has a chord, that is, an edge that connects two vertices of the cycle that are not adjacent in the cycle [9]. We call a phylogenetic network chordal if its underlying graph is chordal.

Finally, we recall that if a graph *G* can be converted into another graph *G*′ by a sequence of vertex deletions, edge deletions, and suppression of degree-2 vertices, *G*′ is called a topological subgraph of *G* [10]. In the present study, we will consider a restricted version of topological subgraphs. In particular, we call a graph *G*′ a restricted topological subgraph of a graph *G* if *G* can be converted into *G*′ by a sequence of the following operations:

1. Deletion of a leaf (and its incident edge).
2. Suppression of a vertex of degree 2.
3. Deletion of a copy of a multiple edge, that is, if *e*_1_ = *e*_2_ ∈ *E*(*G*), then *e*_2_ is deleted.
4. Deletion of a loop, that is, if *e* = {*u*, *u*} ∈ *E*(*G*), then *e* is deleted.

We note that in this case, *G*′ is also a topological subgraph, as the above operations are restricted versions of the respective operations that lead to topological subgraphs: leaf deletion is a special type of vertex deletion, and the deletions of a multiple edge or of a loop are special types of edge deletions.

Finally, a connected and loopless graph *G* is called a GSP graph if it can be reduced to a single edge, that is, to the complete graph *K*_2_, by only applying operations 1–3, that is, by only deleting leaves, suppressing degree-2 vertices, or deleting parallel edges [11]. Similarly, a connected and loopless graph *G* is called a series-parallel graph (SP graph) if it can be reduced to *K*_2_ by operations 2 and 3, that is, by suppressing degree-2 vertices or deleting parallel edges [11].

Both GSP and SP graphs belong to the class of 2-terminal graphs, as shown by the following definition:

**Definition 1** (adapted from [11])

1. The graph *K*_2_ consisting of two vertices *u* and *v* (called *terminals*) and a single edge {*u*, *v*} is a *primitive GSP graph*.
2. If *G*_1_ and *G*_2_ are two GSP graphs with terminals *u*_1_, *v*_1_ and *u*_2_, *v*_2_, respectively, then the graph obtained by any of the following three operations is a GSP graph:
  - Series composition of *G*_1_ and *G*_2_: identifying *v*_1_ with *u*_2_ and specifying *u*_1_ and *v*_2_ as the terminals of the resulting graph.
  - Parallel composition of *G*_1_ and *G*_2_: identifying *u*_1_ with *u*_2_ and *v*_1_ with *v*_2_, and specifying *u*_1_ and *v*_1_ as the terminals of the resulting graph.
  - Generalized series composition of *G*_1_ and *G*_2_: identifying *v*_1_ with *u*_2_ and specifying *u*_2_ and *v*_2_ as the terminals of the resulting graph.

Now, the family of SP graphs consists of those GSP graphs that are obtained using only the series (a) and parallel (b) compositions of Definition 1.

In fact, there is a close relationship between GSP and SP graphs, which is reflected in the following lemma:

**Lemma 1** (adapted from Lemma 3.2 in [11])

A connected graph G is a GSP graph if and only if each block of G (i.e. each maximal induced biconnected subgraph of G) is an SP graph.

## Results

### Reducing phylogenetic networks to related graphs

In the following, we will introduce three methods for reducing phylogenetic networks to related simple graphs, which will play a crucial role in what follows.

#### Leaf cutting

**Fig. 2:**
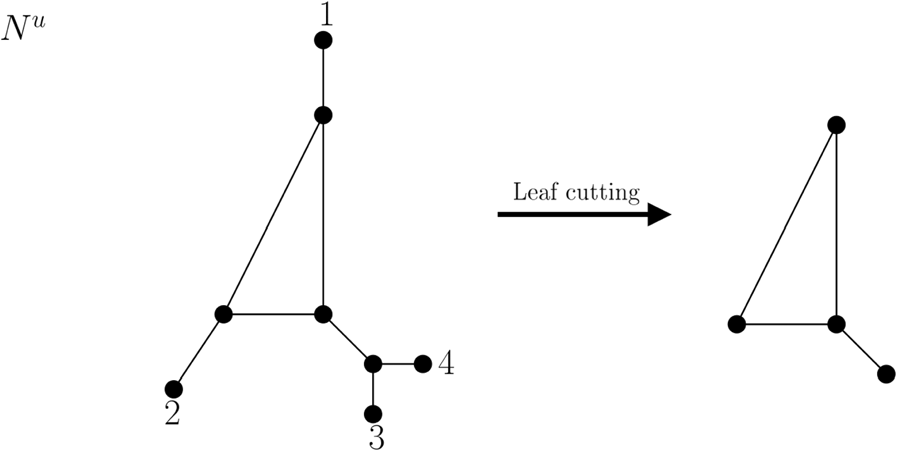
Network N^u^ on labelset X = {1,2,3,4} and the simple graph resulting from the leaf cutting procedure. Note that this procedure results in one new leaf not labeled by X

Let *N*^*u*^ be a phylogenetic network on a taxon set *X* with at least two vertices, at least two of which are leaves, that is, |*V*(*N*^*u*^)| ≥ 2, |*X*| ≥ 2. Let *G* be the simple graph obtained by deleting all leaves labeled by *X* from *V*(*N*^*u*^) and their incident edges; we note that this may result in some vertices of degree 2 and (e.g., if *N*^*u*^ is a tree) even in new leaves not labeled by *X*, which we do not remove. We call the simple graph obtained by this procedure the *leaf cut graph of N*^*u*^ and denote it by $\mathcal{LCUT}\left({N}^u\right)$. An illustration of the described procedure is shown in Fig. 2.

Based on the leaf cutting procedure, we can define a special class of phylogenetic networks, namely, $\mathcal{H}$*-connected* networks, which will be of interest later on.

**Definition 2** Let *N*^*u*^ be a proper phylogenetic network on leaf set *X* with |*X*| ≥ 2 such that $\mathcal{LCUT}\left({N}^u\right)$ is Hamilton connected. Then, *N*^*u*^ is called a $\mathcal{H}$*-connected* network.

We now consider another network reduction procedure, namely, leaf shrinking. We will apply this procedure not only to phylogenetic networks but also to more general connected graphs; thus, we directly define it for general graphs.

#### Leaf shrinking

Let *G* be a connected graph with at least two vertices, at least two of which are leaves, i.e., |*V*(*G*)| ≥ 2, |*V*_*L*_(*G*)| ≥ 2 (where *V*_*L*_(*G*) denotes the set of degree-1 vertices of *G*). We shrink *G* to a smaller simple graph by constructing restricted topological subgraphs as described in Section Methods; that is, we delete vertices of degree 1, suppress vertices of degree 2, and delete a copy of parallel edges or loops. This is performed as follows:

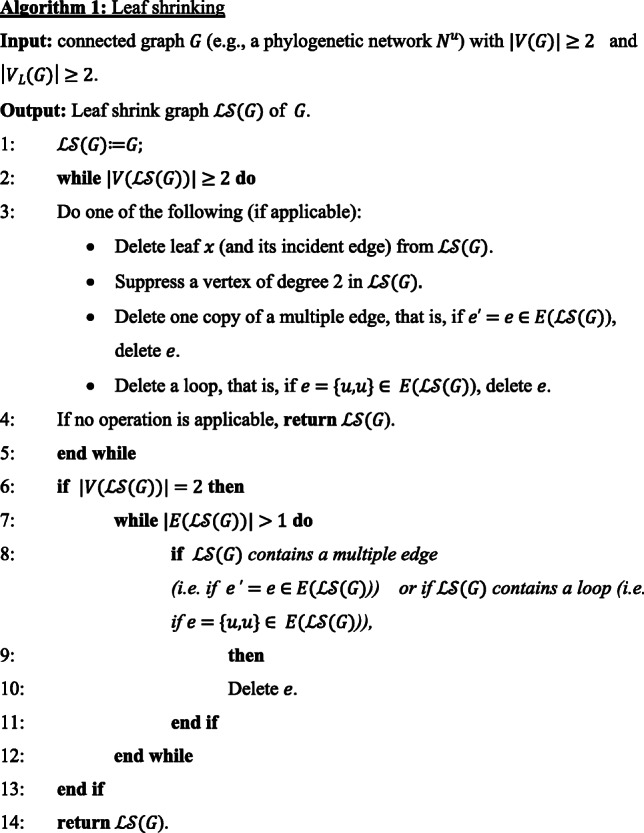



**Fig. 3:**
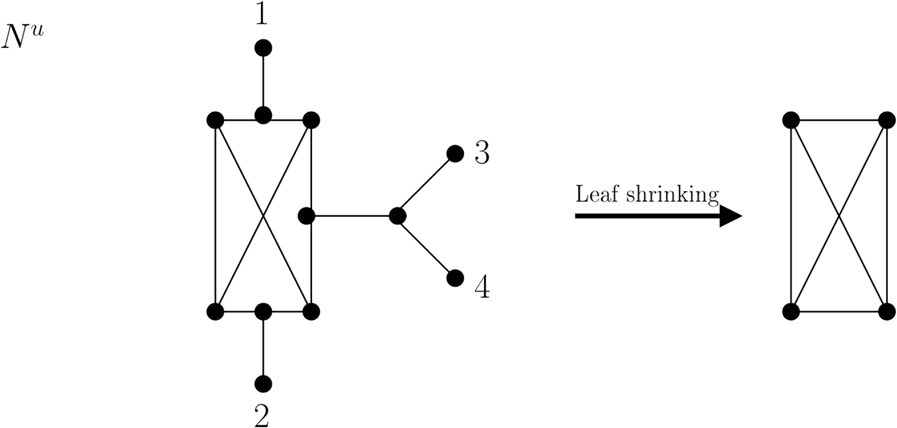
Network N^u^ on labelset X = {1,2,3,4} and the simple graph resulting from the leaf shrinking procedure. At first, leaves 1, 2, 3, and 4 are deleted, resulting in a graph with one new leaf without label, which is subsequently removed. Afterwards, all resulting degree-2 vertices are suppressed

**Fig. 4:**
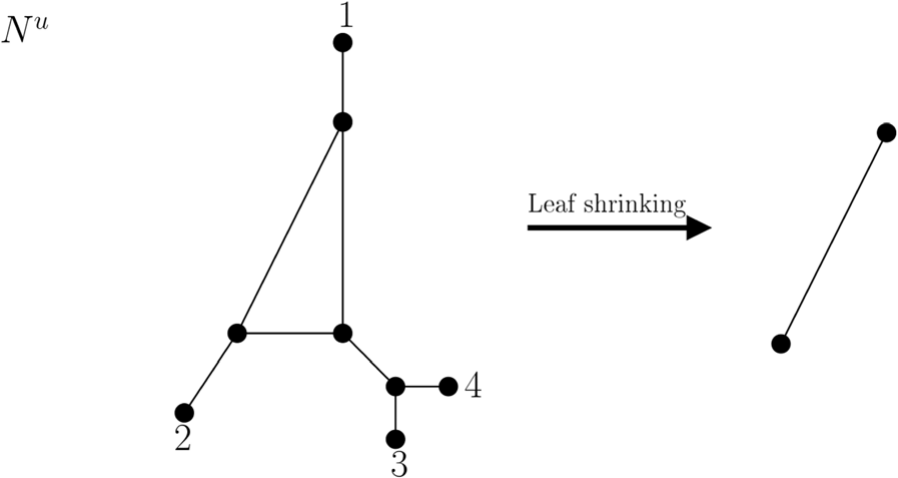
Network N^u^ on labelset X = {1,2,3,4} and the simple graph resulting from the leaf shrinking procedure, which is an edge. At first, leaves 1,2,3 and 4 are deleted, resulting in a graph with one new leaf without label (cf. Fig. 2). Then, this new leaf is removed as well, which results in a triangle. Now, one vertex of degree 2 is suppressed and the parallel edge is deleted resulting in one single edge. Thus, N^u^ is called edge-based. Note that this graph resulting from the leaf shrinking procedure differs from the graph resulting from the leaf cutting procedure depicted in Fig. 2

We call the simple graph obtained by this procedure the *leaf shrink graph of G* and denote it by $\mathcal{LS}(G)$. This notation leads to no ambiguity because we will show in Theorem 2 that $\mathcal{LS}(G)$ is unique. We note that by steps 6–13 in Algorithm 1, the smallest graph (in terms of the number of vertices and the number of edges) to which a graph *G* may be reduced is the complete graph on 2 vertices *K*_2_, that is, a single edge (Fig. 3 and Fig. 4).

Based on the leaf shrinking procedure, we can again introduce a special class of phylogenetic networks, namely, edge-based phylogenetic networks (Fig. 4). We will elaborate on edge-based phylogenetic networks subsequently.

**Definition 3** Let *G* be a connected graph with |*V*(*G*)| ≥ 2 and |*V*_*L*_(*G*)| ≥ 2. If the leaf shrink graph $\mathcal{LS}(G)$ of *G* is a single edge, *G* is called *edge-based.* Else, *G* is called *non-edge-based*. If *G* = *N*^*u*^ is a proper phylogenetic network with |*V*(*N*^*u*^)| ≥ 2 and |*X*| ≥ 2 and $\mathcal{LS}\left({N}^u\right)$ is a single edge, we call *N*^*u*^ an *edge-based* network. Else, *N*^*u*^ is called non-edge-based.

*Remark 1* We note that the definition of edge-based graphs is highly similar to that of GSP graphs; the only difference is that a fourth operation–the deletion of loops–is allowed. However, subsequently, we will show that there is a direct relationship between these two classes of graphs.

The last network reduction procedure that we want to introduce is the so-called leaf connecting procedure.

#### Leaf connecting

**Fig. 5:**
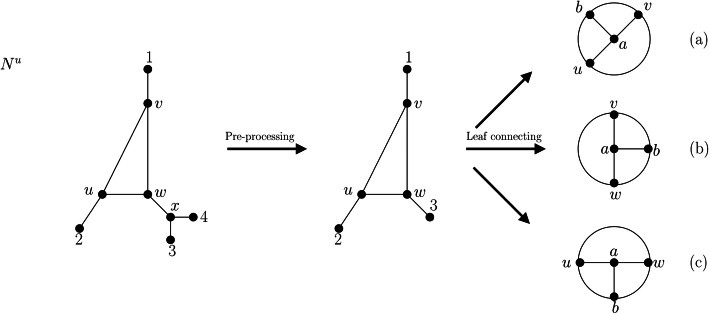
Network *N*^*u*^ and the simple graph resulting from the leaf connecting procedure. First, according to the pre-processing phase of the leaf connecting procedure, leaf 4 is deleted from the network because x is adjacent to two leaves. Then, x has degree 2 and thus needs to be suppressed. Then, first a pair of leaves is chosen and removed from the network, before the last leaf is removed (for (**a**), first leaves 1 and 2 are removed, followed by leaf 3; for (**b**), first leaves 1 and 3 are removed, followed by leaf 2 and for (**c**), first leaves 2 and 3 are removed, followed by leaf 1). Note that the graphs depicted in (**a**), (**b**) and (**c**) are isomorphic. Thus, here $\mathcal{LCON}\left({N}^u\right)$ consists of a single simple graph (in general, $\mathcal{LCON}$ can consist of several simple graphs; as an example see Fig. 6). Note, however, that the simple graph in $\mathcal{LCON}\left({N}^u\right)$ differs from the simple graphs obtained from the leaf cutting and leaf shrinking procedures (cf. Fig. 2 and Fig. 4). Moreover, note that even though new vertices (*a* and *b*) were introduced, the total number of vertices of the simple graph in $\mathcal{LCON}\left({N}^u\right)$ did not increase compared to *N*^*u*^ or even compared to *N*^*u*^ after the pre-processing step

Let *N*^*u*^ be a phylogenetic network that is not a tree^1^ on a taxon set *X* with at least two leaves, that is, |*X*| ≥ 2. Then, we transform *N*^*u*^ into a simple graph without vertices of degree 1 as follows: First, as a pre-processing step, if there exists an internal vertex *v* of *N*^*u*^ such that there is more than one leaf attached to *v*, we delete all but one of the leaves adjacent to *v*. If this results in deg(*v*) = 2, we suppress *v*. We note that this can only occur if *v* is adjacent to only one internal vertex of *N*^*u*^ and at least two leaves. In particular, this implies that suppressing *v* cannot lead to parallel edges (see Fig. 5, where in the pre-processing step, vertex *x* is suppressed).

We note that this pre-processing step may be required to be repeated several times, but this does not affect tree-basedness. If a network is tree-based, there exists a base tree that, in particular, covers all leaves attached to some vertex *v*. By deleting all but one of them and suppressing the resulting degree-2 vertices, we obtain a base tree for the pre-processed network. Conversely, given a base tree for a pre-processed network, we can obtain a base tree for the original network by subdividing edges (if necessary) and adding leaves to these attachment points or to existing vertices of the base tree.

After the pre-processing step, we continue as follows:

- We select two leaves *x*_1_ and *x*_2_ (if they exist). We call their respective attachment points *u*_1_ and *u*_2_, respectively. We delete *x*_1_ and *x*_2_ as well as edges {*x*_1_, *u*_1_} and {*x*_2_, *u*_2_} and add an edge *e* := {*u*_1_, *u*_2_}. If this edge is a parallel edge, that is, if there is another edge *e*′ connecting *u*_1_ and *u*_2_, we add two more vertices *a* and *b* and replace *e* by two new edges, namely *e*_1_ := {*u*_1_, *a*} and *e*_2_ := {*a*, *u*_2_}. Similarly, we replace *e*′ by two new edges, namely, ${e}_1^{\prime}:= \left\{{u}_1,b\right\}$ and ${e}_2^{\prime}:= \left\{b,{u}_2\right\}$. Finally, we add a new edge {*a*, *b*}. We repeat this procedure until no pair of leaves is left.
- If there is one more leaf *x* left, we remove *x*, and if its attachment point *u* then has degree 2, we suppress *u*. If this results in two parallel edges *e* = {*y*, *z*} and *e*′ = {*y*, *z*}, we re-introduce *u* on edge *e*, add a new vertex *a* to the graph, delete *e*′, and introduce two new edges ${e}_1^{\prime}:= \left\{y,a\right\}$ and ${e}_2^{\prime}:= \left\{a,z\right\}$. Finally, we add an edge {*u*, *a*}.

**Fig. 6:**
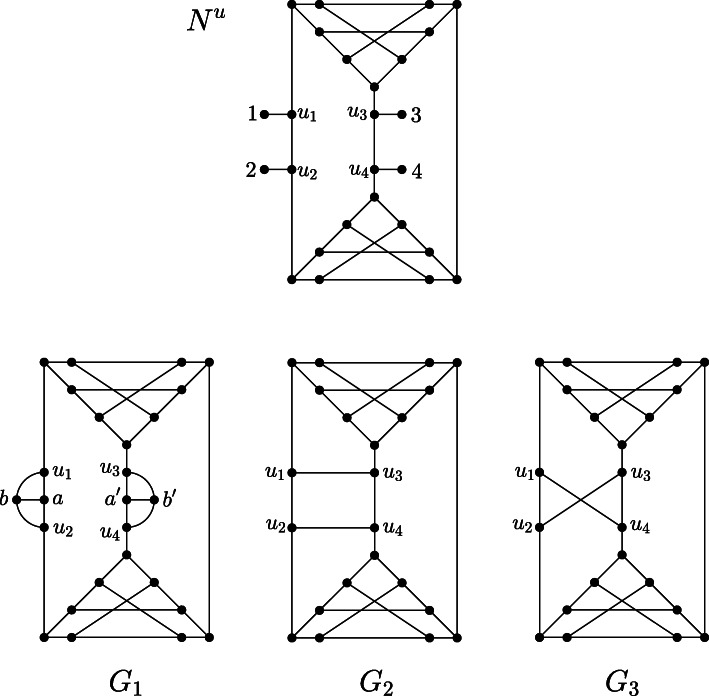
Network *N*^*u*^ (adapted from [5]) and the set $\mathcal{LCON}\left({N}^u\right)$ resulting from the leaf connecting procedure. G_1_ is obtained by deleting leaves 1,2 and 3,4 and connecting their attachment points respectively, while G_2_ is obtained by connecting leaves 1,3 and 2,4 and G_3_ is obtained by connecting leaves 1,4 and 2,3. Note that in case of G_1_, 4 vertices (a,b,a’,b’) have to be introduced in order to prevent the graph from becoming a multigraph. For G_2_ and G_3_ this step is not necessary. Note, however, in any case the number of vertices of a graph in $\mathcal{LCON}\left({N}^u\right)$ cannot increase compared to *N*^*u*^, because in each step 2 leaves are deleted and at most 2 new vertices are created

We note that the order in which the leaves are joined may alter the resulting graph. Thus, if |*X*| > 2, there may be more than one graph that can be obtained from *N*^*u*^ in this manner. We refer to the set of these graphs as $\mathcal{LCON}\left({N}^u\right)$. Two illustrations of this concept are shown in Fig. 5 and Fig. 6.

To summarize, leaf cutting, shrinking and connecting are three different procedures for reducing a phylogenetic network to related simple graphs. In general, the resulting graphs differ. However, all of them lead to sufficient criteria for tree-basedness, which will be introduced in the following. We begin by considering the class of edge-based phylogenetic networks in more detail.

### Classes of tree-based networks

Determining whether an unrooted phylogenetic network is tree-based is generally NP-complete [2]. Accordingly, for practical purposes, it would be useful to know some sufficient properties that can be verified in polynomial time and ensure that a given network is indeed tree-based (even if these criteria are not necessary). In this section, we will introduce a class of tree-based unrooted phylogenetic networks, namely, edge-based networks. Even tough edge-basedness can be verified in linear time, we will additionally mention other classes of networks which are also guaranteed to be tree-based, but are based on properties like being Hamiltonian or Hamilton connected. Although these properties are difficult to verify [12], they have been extensively studied in the context of classical graph theory. Thus, they link phylogenetic network theory to classical graph theory. Moreover, various graphs are already known to be Hamiltonian or Hamilton connected [13–17]. Therefore, these properties may help to further enhance the understanding of phylogenetic networks.

#### Edge-based networks

In this section, we thoroughly analyze the class of edge-based graphs and networks. Our aim is to show that edge-basedness ensures tree-basedness. However, we first show that there is a direct relationship between loopless edge-based graphs and GSP graphs. We then show that the order of the restriction operations is irrelevant for both of them in the following sense: If a graph *G* is edge-based (or GSP), not only does there exist a sequence of restriction operations that reduces *G* to *K*_2_, but also any sequence of restriction operations will lead to a graph on two vertices that can then be further reduced to *K*_2_ (Algorithm 1). Finally, we return to the phylogenetic setting and show that edge-based networks are always tree-based.

### Relationship between edge-based graphs and GSP graphs

By comparing the definitions of GSP graphs and edge-based graphs a slight difference between the two classes is observed. Specifically, both can be reduced to a single edge by certain restriction operations; however, loop deletion is a valid restriction operation in the case of edge-based graphs, but not in the case of GSP graphs. Nevertheless, in the following, we will show that there is a direct relationship between both classes of graphs.

**Theorem 1** *Let* *G* *be a connected graph. Then* *G* *is a GSP graph if and only if*

(i) *G is loopless and*
(ii) *G can be reduced to K*_2_ *by deleting leaves, suppressing vertices of degree 2, deleting copies of parallel edges and deleting loops, that is, by applying restriction operations 1–4 (Section **Further graph-theoretical concepts**)*.

*Proof* First, we assume that *G* is a GSP graph. Then, by definition, *G* does not contain loops, that is, (i) holds. Moreover, *G* can be reduced to *K*_2_ by applying restriction operations 1–3 (p. 3), and thus (ii) holds as well.

**Fig. 7:**
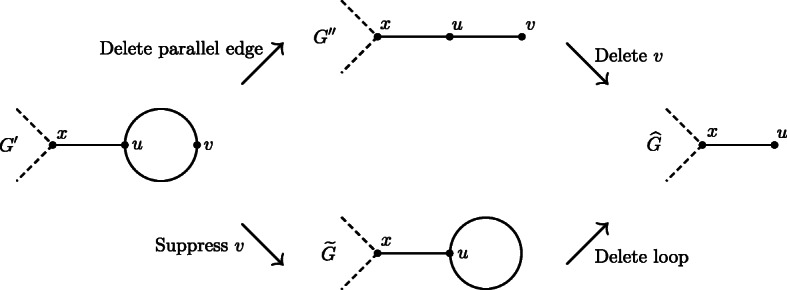
Two alternative ways to reach graph G ^ from G’ (and thus from G)

We now assume that *G* is a connected graph without loops that can be reduced to *K*_2_ by applying restriction operations 1–4: To show that *G* is a GSP graph, we should show that *G* can also be reduced to *K*_2_ by only applying operations 1–3, that is, by deleting leaves, suppressing degree-2 vertices, and deleting copies of parallel edges, but not deleting loops. As *G* is by assumption a graph without loops, loops can only arise during the reduction process. Let $\overset{\sim }{G}$ be a restricted topological subgraph of *G* that contains a loop. We assume that $\overset{\sim }{G}$ is the first graph with loops that arises when *G* is reduced to *K*_2_. This implies that in the transformation of *G* into $\overset{\sim }{G}$, there must have been a restricted topological subgraph *G*′ of *G* containing a parallel edge *e* = {*u*, *v*}, where one of *u* and *v* (without loss of generality, *v*) was a degree-2 vertex, and the step from *G*′ to $\overset{\sim }{G}$ was the suppression of *v*. Then, deleting the loop {*u*, *u*} from $\overset{\sim }{G}$ yields some restricted topological subgraph $\hat{G}$ of *G*. However, $\hat{G}$ can alternatively be reached from *G*′ by first deleting a copy of the parallel edge *e* = {*u*, *v*} (yielding a graph *G*′′) and then deleting vertex *v*. Thus, $\hat{G}$ can be obtained from *G* by only applying operations 1–3 (Fig. 7). As the deletion of loops can always be circumvented in this manner, *G* in particular can be reduced to *K*_2_ by only applying operations 1–3. Together with the fact that *G* is loopless, this implies that *G* is a GSP graph. This completes the proof.

As the following corollary shows, Theorem 1 implies that there is a one-to-one correspondence between loopless edge-based graphs and GSP graphs.

**Corollary 1** *Let G be a connected graph. Then G is a GSP graph if and only if it is loopless and edge-based*.

*Proof* We first assume that *G* is a GSP graph. Then, by Theorem 1, *G* is loopless and can be reduced to *K*_2_ by deleting leaves, suppressing degree-2 vertices, deleting copies of parallel edges and deleting loops. Let $\hat{G}$ be a restricted topological subgraph of *G* with $\left|V\left(\hat{G}\right)\right|=2$. Then, either $\hat{G}={K}_2$ or $\hat{G}$ can be reduced to *K*_2_. However, the latter reduction cannot require the deletion of leaves or suppression of degree-2 vertices (as this would reduce the number of vertices to less than 2, and then *K*_2_ could not be a restricted topological subgraph). This implies that *G* can be reduced to *K*_2_ by applying Algorithm 1, and thus *G* is edge-based.

We now assume that *G* is loopless and edge-based. The latter implies that *G* can be reduced to *K*_2_ by applying Algorithm 1. Together with Theorem 1 and the fact that *G* is loopless, the implication is that *G* is a GSP graph, which completes the proof.

We note that GSP graphs can be recognized in linear time [11, 18]. A naïve approach would be, for example, to consider the maximal biconnected components (or blocks) of a graph *G*, which can be computed in linear time [19], and use the fact that a graph *G* is GSP if and only if each block of *G* is an SP graph (Lemma 1), which can again be recognized in linear time [20]. Owing to the one-to-one correspondence between GSP graphs and loopless edge-based graphs, this implies that edge-basedness can also be tested in linear time. In particular, it can be determined in linear time whether an unrooted phylogenetic network is edge-based. As we will later show that edge-basedness implies tree-basedness (Theorem 3), this is of great relevance because, in general, the problem of determining whether a network is tree-based is NP-complete [2].

However, before analyzing the relationship between edge-basedness and tree-basedness, we first state another interesting property of edge-based and GSP graphs, namely, that the order of the restriction operations is irrelevant.

### Order of restriction operations

**Theorem 2** *Let G be a graph. Then,* $\mathcal{LS}(G)$ *is unique. In particular, if G is an edge-based graph, all sequences of restriction operations in concordance with Algorithm 1 lead to K*_2_.

*Remark 2* Theorem 2 implies that the order of the restriction operations is irrelevant provided that the rules of Algorithm 1 are followed, that is, if two or more operations are possible, it is irrelevant which is chosen. However, we recall that if $\mid V\Big(\mathcal{LS}(G)\mid =2$, the choice of the restriction operation is limited to deleting copies of parallel edges or deleting loops to prevent the number of vertices from dropping below 2.

The proof of Theorem 2 requires the following lemmas.

**Lemma 2** *Let G be a graph with vertex set V*(*G*) *and edge set E*(*G*) *such that* *G* *has some graph* *H* *as a restricted topological subgraph. Let G*′ *result from G by precisely one of the following operations*:

1. *Choose a vertex u* ∈ *V*(*G*)*, introduce a new vertex x and an edge* {*u*, *x*} *(‘Add leaf x ’).*
2. *Choose an edge e* ∈ *E*(*G*) *and subdivide it into two edges by introducing a new degree-2 vertex (‘Add a degree-2 vertex’).*
3. *Choose an edge e* ∈ *E*(*G*) *and add a copy e*′ *of e to E*(*G*)*.*
4. *Choose a vertex u* ∈ *E*(*G*) *and add a loop,* i.e.*, add edge e* = {*u*, *u*} *to E*(*G*)*.*

*Then, H* *is also a restricted topological subgraph of G*′*.*

*Proof* We can convert *G*′ into *G* by undoing the respective operation. Then, as *G* can be reduced to *H*, so can *G*′ (using the conversion to *G* as a first step and adding the sequence that converts *G* to *H*). This completes the proof.

The proofs of the following two lemmas can be found in Appendix.

**Lemma 3** *Let G be a connected graph with vertex set V(G) and edge set E(G). Let G′ result from G by deleting one loop. Then, a graph H (with H ≠ G) is a restricted topological subgraph of G if and only if H is a restricted topological subgraph of G′.*

**Lemma 4** *Let G be a connected graph with vertex set V(G) and edge set E(G). Let G′ result from G by deleting one copy of a parallel edge. Then, a graph H (with H ≠ G) is a restricted topological subgraph of G if and only if H is a restricted topological subgraph of G*′.

The last two lemmas immediately imply the following corollary, which plays a fundamental role in the proof of Theorem 2.

**Corollary 2** *Let G be a graph and let G′ be its underlying simple graph. Moreover, let H be a graph with* $\mathcal{LS}(H)$ = *H (that is, H cannot be reduced to a graph H′ ≠ H by Algorithm 1). Then, H is a restricted topological subgraph of G if and only if H is a restricted topological subgraph of G′*.

*Proof G*′ has the same structure as *G* but without parallel edges and loops. If *G*′ has *H* as a restricted topological subgraph, by repeatedly applying operations 3 and 4 of Lemma 2, so does *G*. If *G* has *H* as a restricted topological subgraph, by repeatedly applying Lemma 3 and Lemma 4, so does *G*′. This completes the proof.

We are finally in a position to prove Theorem 2.

*Proof (Theorem 2)* Let *G* be a graph with leaf shrink graph *H*, and we assume that *LS*(*G*) is not unique, that is, we assume that *G* also has a leaf shrink graph *H'* with *H ≠ H'*. More precisely, we assume that there exists a sequence *σ* of restriction operations as in Algorithm 1 that does not lead to *H*, but to *H'*. This implies that *G* has *H* as a restricted topological subgraph, but it also has some restricted topological subgraph that does not have *H* as a restricted topological subgraph (as *σ* does not lead to *H*).

We consider a minimal graph with this property in terms of the number of vertices. Thus, we assume that *G* has *H* as a restricted topological subgraph, but there exists a restricted topological subgraph *G*′ of *G* that does not have *H* as a restricted topological subgraph, and there is no other graph with this property containing fewer vertices than *G*. By Corollary 2, we may assume that *G* has no loops and no parallel edges.

We now consider the reduction of *G* to *G*′. As *G* has no parallel edges and no loops, the first step in the transformation of *G* into *G*′ must be the deletion of a leaf or the suppression of a degree-2 vertex. Moreover, the resulting graph *G*′′ after one step must already be such that *H* is not a restricted topological subgraph; otherwise, *G*′′ would also have *G*′ as a restricted topological subgraph (as it is on the path from *G* to *G*′), it would have *H* as a restricted topological subgraph, and it would have strictly fewer vertices than *G*, which would contradict the minimality of *G*.

Let us now consider *G*′′. Then, *G*′′ can be arrived at from *G* by deleting a leaf *x* or suppressing a vertex *u* of degree 2, and *H* is a restricted topological subgraph of *G* but not of *G*′′. Moreover, we consider $\overset{\sim }{G}$, which shall be a graph that can be obtained from *G* at one step (i.e., after one restriction operation) in the transformation of *G* into *H*. As $\overset{\sim }{G}$ has *H* as a restricted topological subgraph, and as $\overset{\sim }{G}$ has strictly fewer vertices than *G*, we know that *all* restricted subgraphs of $\overset{\sim }{G}$ have *H* as a restricted topological subgraph.

We now consider the case that a leaf *x* has been deleted in the transformation of *G* into *G*′′. We note that *x* is also present in $\overset{\sim }{G}$, as *x* cannot be affected by any restriction operation other than the deletion of *x* (*G*′′ and $\overset{\sim }{G}$ cannot be equal and both differ from *G* by the removal of precisely one vertex). Thus, we now delete *x* from $\overset{\sim }{G}$ to obtain a graph $\hat{G}$ that has *H* as a restricted topological subgraph. By Lemma 2, we can undo the step that has been performed in the transformation of *G* into $\overset{\sim }{G}$, that is, we can re-add to $\hat{G\ }$ the leaf that has been deleted or the suppressed degree-2 vertex, and the resulting graph (which is precisely *G*′′) has *H* as a restricted topological subgraph. This contradicts the construction of *G*′′.

If now a degree-2 vertex *u* has been suppressed in the transformation of *G* into *G*′′, then either *u* is still present as a degree-2 vertex in $\overset{\sim }{G}$, or *u* is a leaf in $\overset{\sim }{G}$ (if a leaf adjacent to *u* has been deleted). In the former case, that is, if *u* still has degree 2 in $\overset{\sim }{G}$, we can suppress *u* to obtain a graph $\hat{G}$ that has *H* as a restricted topological subgraph. By Lemma 2, we can undo the step that has been performed in the transformation of *G* into $\overset{\sim }{G}$, that is, we can re-add to $\hat{G}$ the leaf that has been deleted or the suppressed degree-2 vertex, and the resulting graph, which is precisely *G*′′, has *H* as a restricted topological subgraph. This contradicts the construction of *G*′′.

**Fig. 8:**
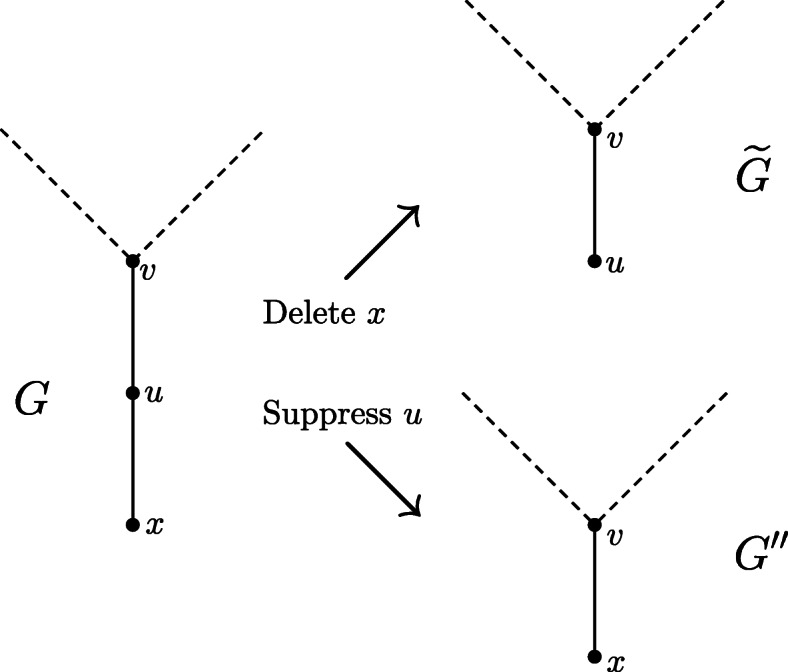
Two isomorphic graphs G ~ and G” that are constructed by either deleting leaf x or suppressing vertex u

Thus, the only remaining case is when a degree-2 vertex *u* has been suppressed in the transformation of *G* into *G*′′, and *u* is a leaf in $\overset{\sim }{G}$. However, this can occur only if a leaf *x* adjacent to *u* has been deleted in the transformation of *G* into $\overset{\sim }{G}$, and if *u* is a degree-2 vertex adjacent to a leaf, then deleting the leaf and its incident edge is equivalent to suppressing *u*, that is, the resulting graphs *G*′′ and $\overset{\sim }{G}$ are isomorphic. This is illustrated by Fig. 8. Thus, as *H* is a restricted topological subgraph of $\overset{\sim }{G}$, it is also a restricted topological subgraph of *G*′′, but this contradicts the construction of *G*′′.

Therefore, all cases lead to a contradiction, which shows that the initial assumption is false. In particular, all sequences of restriction operations as in Algorithm 1 eventually lead to *H*. This completes the proof.

### Edge-basedness implies tree-basedness

We now state the last main theorem of this section, which shows that all edge-based networks (Definition 3) are also tree-based.

**Theorem 3** *Let N*^*u*^ *be a proper phylogenetic network on leaf set X with* |*X*| ≥ 2*. If N*^*u*^ *is edge-based, it is also tree-based.*

We note that the converse does not hold: Fig. 3 shows a tree-based network *N*^*u*^ that is not edge-based.

To prove Theorem 3, we will exploit the one-to-one correspondence between loopless edge-based graphs and GSP graphs (Corollary 1). Moreover, we will use the fact that a graph is GSP if and only if its blocks are SP graphs (Lemma 1).

The strategy for the proof of Theorem 3 is thus to decompose an edge-based network *N*^*u*^ into its blocks (which are SP graphs by Lemma 1, as *N*^*u*^ is loopless by definition and hence a GSP graph by Corollary 1), obtain a certain spanning tree for each block, and use these spanning trees to construct a support tree for *N*^*u*^. This requires the following additional technical lemma, the proof of which is given in Appendix.

**Lemma 5** *Let G = (V, E) be a simple and biconnected SP graph with at least three vertices. Then, there exists a spanning tree T in G whose leaves correspond to the degree-2 vertices of G. In particular, no vertex v ∈ V (G) with deg (v) > 2 is a leaf in T.*

*Remark 3* In the following, given a simple and biconnected SP graph *G* with at least three vertices, we call a spanning tree *T* having only degree-2 vertices of *G* as leaves a valid spanning tree. Additionally, given the trivial SP graph *K*_2_, we also call a spanning tree for *K*_2_ (which is *K*_2_ itself) a valid spanning tree.

With this we are now in a position to prove Theorem 3.

*Proof of Theorem 3* Let *N*^*u*^ be a proper phylogenetic network on a leaf set *X* with |*X*| ≥ 2. If |*V*(*N*^*u*^)| = |*X*| = 2 and *N*^*u*^ consists of a single edge, *N*^*u*^ is trivially tree-based. Thus, we may assume that |*V*(*N*^*u*^)| ≥ 3.

**Fig. 9:**
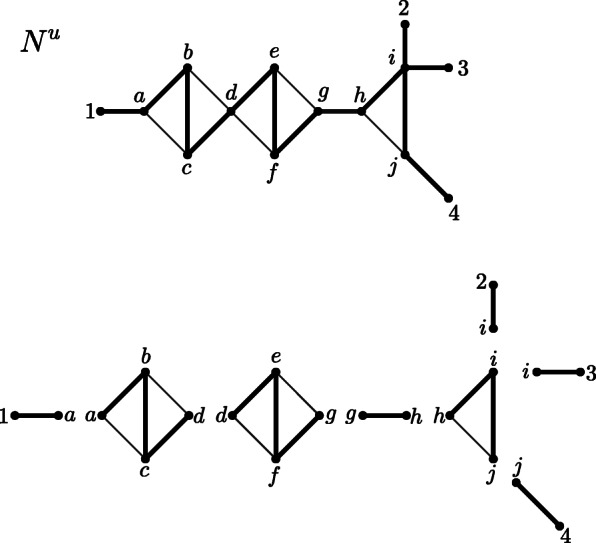
Decomposition of the edge-based network N^u^ into its maximal biconnected components that are either trivial SP graphs, i.e., single edges (corresponding to cut edges of N^u^), or simple and biconnected SP graphs with at least three vertices. For the latter, valid spanning trees are depicted in bold, respectively. The edges of these spanning trees together with all cut edges (also depicted in bold) yield a support tree for N^u^ and thus N^u^ is tree-based

As *N*^*u*^ is edge-based and loopless, it is a GSP graph by Corollary 1, and we can decompose it into its blocks, that is, into its maximal biconnected components (Fig. 9). By Lemma 1, these blocks are SP graphs. More precisely, each block of *N*^*u*^ is either a trivial SP graph (i.e., a single edge corresponding to a cut edge of *N*^*u*^) or a simple and biconnected SP graph with at least three vertices.

We now consider all blocks $\mathcal{B}$ of *N*^*u*^ and construct a support tree *T* for *N*^*u*^ as follows:

If $\mathcal{B}=\left\{u,v\right\}$ is a single edge (i.e., $\mathcal{B}$ is a cut edge of *N*^*u*^), we add this edge to *T*, whereas if $\mathcal{B}$ is a simple and biconnected SP graph with at least three vertices, we add all edges of a valid spanning tree ${T}_{\mathcal{B}}$ of $\mathcal{B}$ (i.e., of a spanning tree for $\mathcal{B}$ having only degree-2 vertices of $\mathcal{B}$ as leaves, which must exist by Lemma 5), to *T*.

Then, *T* is a support tree for *N*^*u*^ because:

- *T* covers all vertices of *N*^*u*^ (as it covers all vertices of each block $\mathcal{B}$ of *N*^*u*^).
- *T* is a tree, that is, *T* is connected and acyclic. To see this, we note that any two blocks ${\mathcal{B}}_1$ and ${\mathcal{B}}_2$ of *N*^*u*^ share at most one common vertex, which is a cut vertex of *N*^*u*^. Let ${T}_{{\mathcal{B}}_1}$ be a valid spanning tree of ${\mathcal{B}}_1$ and let ${T}_{{\mathcal{B}}_2}$ be a valid spanning tree for ${\mathcal{B}}_2$ (where both ${T}_{{\mathcal{B}}_1}$ and ${T}_{{\mathcal{B}}_2}$ are potentially single edges). Further, we assume that ${\mathcal{B}}_1$ and ${\mathcal{B}}_2$ share a common vertex *v*. Then identifying the copy of *v* in ${T}_{{\mathcal{B}}_1}$ with the copy of *v* in ${T}_{{\mathcal{B}}_2}$ yields a spanning tree for ${\mathcal{B}}_1\cup {\mathcal{B}}_2$, as identifying the two copies of *v* cannot induce cycles because ${\mathcal{B}}_1$ and ${\mathcal{B}}_2$ (and thus ${T}_{{\mathcal{B}}_1}$ and ${T}_{{\mathcal{B}}_2}\Big)$ do not share any vertices other than *v*. As every block of *N*^*u*^ contains at least one cut vertex of *N*^*u*^ and as *T* covers all cut vertices of *N*^*u*^, it iteratively follows that *T* is connected and acyclic.
- The leaf set of *T* corresponds to *X*. To see this, we consider the leaves of the induced spanning trees ${T}_{\mathcal{B}}$ for each block $\mathcal{B}$ of *N*^*u*^.
  - If $\mathcal{B}$ is a non-trivial SP graph, its valid spanning tree ${T}_{\mathcal{B}}$ has only degree-2 vertices of $\mathcal{B}$ as leaves. Let *v* be such a leaf. As *N*^*u*^ does not contain degree-2 vertices (because it is a phylogenetic network), *v* must be a cut vertex of *N*^*u*^. However, by the preceding argument, *v* is then contained in at least one other spanning tree ${T}_{\mathcal{B}\prime }$ for some other block $\mathcal{B}^{\prime }$ of *N*^*u*^ and thus cannot be a leaf in *T* (as in *T*, the two copies of *v* contained in ${T}_{\mathcal{B}}$ and ${T}_{\mathcal{B}\prime }$, respectively, are identified, and thus deg(*v*) ≥ 2 in *T*).
  - Similarly, if $\mathcal{B}$ is a trivial SP graph {*u*, *v*}, and if {*u*, *v*} is an internal cut edge of *N*^*u*^, neither *u* nor *v* can be leaves in *T* (as again, both *u* and *v* are contained in at least one other spanning tree, and after identifying all copies of *u* and all copies of *v*, respectively, we have deg(*u*), deg(*v*) ≥ 2 in *T*).
  - Finally, if $\mathcal{B}=\left\{x,v\right\}$ is a trivial SP graph corresponding to an external cut edge of *N*^*u*^, where *x* ∈ *X* and *v* is an internal vertex of *N*^*u*^, *x* is a leaf in *T* and *v* is an internal vertex in *T*. This is because each leaf *x* of *N*^*u*^ is contained in exactly one block of *N*^*u*^ (and thus, it will be a leaf in *T*, as there is only one copy of *x*), whereas there exists at least one other block $\mathcal{B}^{\prime }$ containing a copy of *v*, and the two copies of *v* will be identified in *T*.

To summarize, *T* is a spanning tree of *N*^*u*^ that contains all leaves *x* ∈ *X* but does not induce any additional leaves. Thus, *T* is a support tree for *N*^*u*^, and *N*^*u*^ is tree-based. This completes the proof.

In conclusion, edge-based networks are always tree-based and, more importantly, whether a network is edge-based can be verified in linear time.

Additionally, we note that to verify the edge-basedness of a network, we can use the fact that a network can be seen as a “blobbed” tree [6], that is, as a tree with blobs as vertices. In particular, we have the following decomposition, which is the final result of this section.

**Proposition 1** *Let N*^*u*^ *be a proper unrooted phylogenetic network with at least two leaves. Then, N*^*u*^ *is edge-based if and only if every non-trivial blob of N*^*u*^ *is edge-based.*

The proof of this proposition again exploits the one-to-one correspondence between loopless edge-based graphs and GSP graphs and uses the following theorem, which implies that a GSP graph can be reduced to any of its edges.^2^

**Theorem 4** (Theorem 4.1 in [11]).

*Let G be a GSP graph. Then, for any edge e* = {*u*, *v*} *of G, G is a GSP graph with terminals u and v.*

We now use this theorem to prove Proposition 1.

*Proof of Proposition 1* We first note that if *N*^*u*^ contains only trivial blobs, it is a tree and is therefore trivially edge-based. Thus, we now consider the case that *N*^*u*^ contains at least one non-trivial blob. If *N*^*u*^ is edge-based, then all non-trivial blobs of *N*^*u*^ are also necessarily edge-based. If there was a non-trivial blob of *N*^*u*^ with a restricted topological subgraph that could not be reduced to an edge, this subgraph would also be contained as a restricted topological subgraph in *N*^*u*^; this implies that *N*^*u*^ would have a restricted topological subgraph that could not be reduced to an edge. However, by Theorem 2, all restricted topological subgraphs of *N*^*u*^ must have a single edge as a restricted topological subgraph, and this is a contradiction.

If all non-trivial blobs of *N*^*u*^ are edge-based, then we can inductively show that *N*^*u*^ is edge-based. If *N*^*u*^ contains only one non-trivial blob, there is nothing to show. We now assume that the statement is true for all networks with at most *m* non-trivial blobs, and let *N*^*u*^ contain *m* + 1 non-trivial blobs. Then, we use the fact that *N*^*u*^ must contain a cut edge *e* = {*a*, *b*} whose removal results in two connected components, each containing at least one non-trivial blob. We denote these components by ${N}_a^u$ and ${N}_b^u$ and assume that *a* is contained in ${N}_a^u$ and *b* is contained in ${N}_b^u$. We now re-introduce the cut edge {*a*, *b*} to both components by attaching a new leaf *a* to ${N}_b^u$ and *b* to ${N}_a^u$. Without loss of generality, we first consider ${N}_a^u$. As ${N}_a^u$ contains at most *m* non-trivial blobs, it is edge-based by the inductive hypothesis. Moreover, by Theorem 4, we can reduce it to any of its edges, in particular, to its leaf edge *e* = {*a*, *b*}.

Similarly, as ${N}_b^u$ contains at most *m* non-trivial blobs, it is also edge-based and can be reduced to its leaf edge *e* = {*a*, *b*}. In total, this implies that *N*^*u*^ can be reduced to edge *e* = {*a*, *b*}. In particular, *N*^*u*^ is edge-based. This completes the proof.

#### Other networks that are necessarily tree-based

After having thoroughly analyzed edge-based networks, we will now consider other classes of networks that are necessarily tree-based by using some classical graph theoretical arguments.

**Theorem 5** *Let N*^*u*^ *be a proper phylogenetic network on leaf set X with* |*X*| ≥ 2*, and consider* $\mathcal{LCUT}\left({N}^u\right)$ *as well as the set* $\mathcal{LCON}\left({N}^u\right)$ *as defined in Section* *Reducing phylogenetic networks to related graphs*. *Then, the following statements hold:*

1. *If N*^*u*^ *contains two leaves x and y with attachment points u and v, respectively, such that the edge* {*u*, *v*} *is contained in the edge set of N*^*u*^ *and such that there is a path in N*^*u*^ *from u to v visiting all inner vertices of N*^*u*^*, then N*^*u*^ *is tree-based.*
2. *If N*^*u*^ *is an* $\mathcal{H}$*-connected network (*i.e.*, if* $\mathcal{LCUT}\left({N}^u\right)$ *is Hamilton connected), then N*^*u*^ *is tree-based.*
3. *If there is a graph G in* $\mathcal{LCON}\left({N}^u\right)$ *such that G is Hamiltonian and contains a Hamiltonian cycle which uses an edge of G which is not contained in N*^*u*^ *and which did not result from deleting the last leaf in case* ∣*X*^*r*^∣ *is odd (where X*^*r*^ *denotes the reduced leaf set of N*^*u*^ *after a potential pre-processing step), then N*^*u*^ *is tree-based.*
4. *If there is a graph G in* $\mathcal{LCON}\left({N}^u\right)$ *such that G is Hamiltonian and such that at least two new vertices, say a and b, had to be added when connecting the attachment points u and v of two leaves x and y during the construction of G in order to prevent parallel edges, then N*^*u*^ *is tree-based.*

**Fig. 10:**
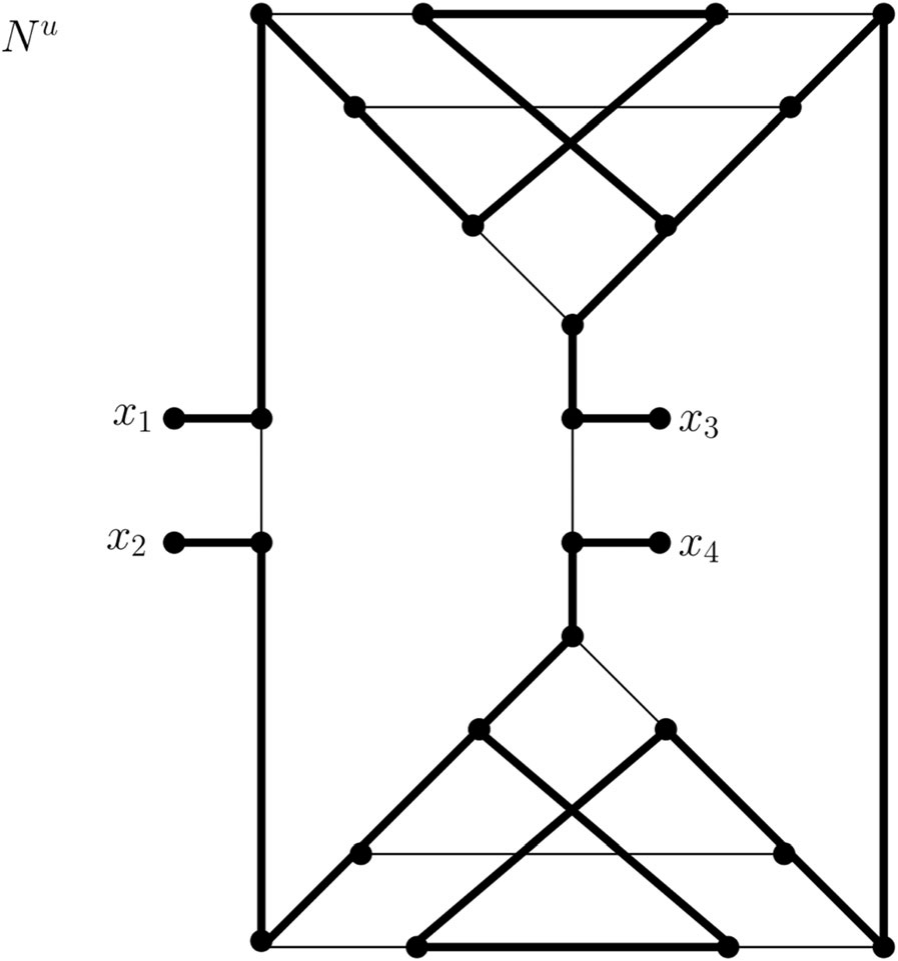
Binary tree-based unrooted phylogenetic network N^u^ on X = {x_1_,x_2_ x_3_,x_4_}. The corresponding support tree is highlighted in bold. N^u^-x_i_ is not tree-based for i = 1, …,4, because there is no spanning tree in N^u^-x_i_ whose leaf set is equal to X∖{x_i_} (Figure taken from [5])

We note that the converse of this theorem does not hold. Fig 10 demonstrates that the converse of the first part of Theorem 5 does not hold, as it depicts a tree-based network that does not contain a path from one attachment point of a leaf to any other and visits all inner vertices. Such a path would imply a Hamiltonian path from one leaf to another (when the remaining leaves are disregarded), which does not exist.

Moreover, Fig. 2 shows an example of a tree-based network for which $\mathcal{LCUT}\left({N}^u\right)$ is not Hamilton connected. Accordingly, the implication in the second part of Theorem 5 cannot be reversed.

Fig 6 shows an example of a tree-based network for which there is no *G* in $\mathcal{LCON}\left({N}^u\right)$ such that *G* is Hamiltonian. *G*_1_, *G*_2_ and *G*_3_ in $\mathcal{LCON}\left({N}^u\right)$ do not contain a Hamiltonian cycle. Thus, conditions three and four in Theorem 5 are also sufficient but not necessary.

**Fig. 11:**
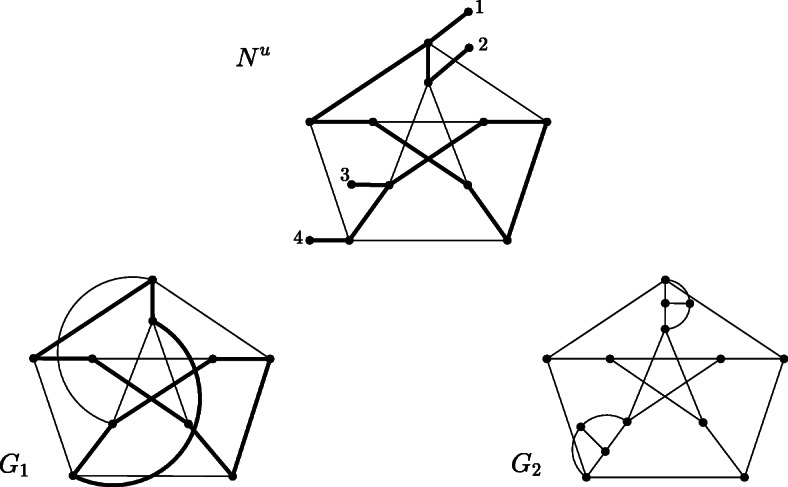
Tree-based network N^u^ (support tree depicted in bold) that is based on the Petersen graph. G_1_ and G_2_ are both in $\mathcal{LCON}\left({N}^u\right)$, but only G_1_ is Hamiltonian (a Hamiltonian cycle is depicted in bold)

Moreover, before proceeding with the proof of the theorem, we mention that concerning $\mathcal{LCON}\left({N}^u\right)$, the exact order in which the leaves are connected can play a fundamental role. Fig 11 shows a tree-based phylogenetic network (based

on the famous Petersen graph), and two different graphs in $\mathcal{LCON}\left({N}^u\right)$. However, only one of them is Hamiltonian, whereas the other is not because the Petersen graph is non-Hamiltonian (see, for example, properties of the Petersen graph in the “House of graphs” database (graph ID 660 [21]);.

We now prove Theorem 5.

*Proof of Theorem 5*

1. If *N*^*u*^ contains two leaves *x* and *y* with attachment points *u* and *v*, respectively, such that the edge {*u*, *v*} is contained in the edge set of *N*^*u*^ and such that there is a path in *N*^*u*^ from *u* to *v* visiting all inner vertices of *N*^*u*^, then we can construct a support tree *T* for *N*^*u*^ as follows: We consider the path from *u* to *v* visiting all inner vertices of *N*^*u*^ and add all leaves of *N*^*u*^ together with their pending edges to it. As all attachment points of leaves are already contained in the path (because this path visits all inner vertices), the re-introduction of all leaves implies that *T* indeed covers all vertices of *N*^*u*^. As we did not add the edge {*u*, *v*}, there is no cycle. In total, *T* is a spanning tree of *N*^*u*^. Moreover, its leaf set must coincide with that of *N*^*u*^: All leaves of *N*^*u*^ are also leaves of *T* (because a degree-1 vertex of *N*^*u*^ naturally has degree 1 in *T* as well). Moreover, all vertices on the path from *u* to *v* have degree at least 2, except for *u* and *v*. However, as *u* and *v* were attachment points of leaves, after their re-attachment, they also have degree at least 2 in *T*. Accordingly, *T* cannot have any leaves that are not leaves of *N*^*u*^. Therefore, *T* is a support tree of *N*^*u*^, and thus *N*^*u*^ is tree-based.
2. Let *N*^*u*^ be a $\mathcal{H}$-connected network, that is, let $\mathcal{LCUT}\left({N}^u\right)$ be Hamilton connected. We consider any two leaves *x* and *y* of *N*^*u*^ and their respective attachment points, *u* and *v*. As $\mathcal{LCUT}\left({N}^u\right)$ is Hamilton connected, there is a Hamiltonian path from *u* to *v* in $\mathcal{LCUT}\left({N}^u\right)$. We now consider this path in *N*^*u*^ and extend it by all pending edges of all leaves. This leads to a tree *T* that covers all inner vertices on the original path from *u* to *v* and all leaves as they were re-attached. There cannot be any cycles, as the Hamiltonian path itself has no cycle, and adding leaves, which are of degree 1, cannot create cycles. Thus, *T* is a spanning tree of *N*^*u*^. Moreover, the leaf set of *T* coincides with that of *N*^*u*^: All vertices on the path from *u* to *v* except for *u* and *v* have degree 2 before the re-attachment of their leaves. *u* and *v* have degree 1 in the path, but their leaves *x* and *y* were also re-attached; thus, in the final tree, they have degree 2. Therefore, the only degree-1 vertices in *T* are the leaves of *N*^*u*^. Accordingly, *T* is a support tree, and thus *N*^*u*^ is tree-based.
3. Let us now assume that there is a *G* in $\mathcal{LCON}\left({N}^u\right)$ such that *G* contains a Hamiltonian cycle that uses at least one of the edges that *N*^*u*^ does not contain (i.e., that were introduced in the transformation of *N*^*u*^ into *G*). We consider such a graph *G* and such a Hamiltonian cycle. We note that as this cycle covers all vertices of *G*, it covers, in particular, all vertices to which the leaves of *N*^*u*^ are attached. Moreover, it covers all vertices of *G* that are not in *N*^*u*^, namely, precisely the vertices of type *a* and *b* that may have been added in the construction of *G* to prevent parallel edges. We will now transform this cycle into a support tree of *N*^*u*^ as follows.

- If no new vertices were added when *G* was constructed, then no connection of leaves led to parallel edges. However, as *N*^*u*^ has at least two leaves, at least one edge of *G* is not an edge of *N*^*u*^. By assumption, such an edge {*u*, *v*} is covered by the Hamiltonian cycle of *G* under consideration. Then, we consider the same cycle in *N*^*u*^ but break the edge {*u*, *v*} to obtain an acyclic tree. This path tree has only two vertices of degree 1, namely *u* and *v*. However, as the edge {*u*, *v*} was added in the construction of *G*, both *u* and *v* are leaf attachment points in *N*^*u*^. We now re-attach all leaves to transform this path tree into a tree *T* so that its only leaves are the leaves of *N*^*u*^ (because the degrees of both *u* and *v* are now at least 2), and, by construction, it covers all vertices of *N*^*u*^. Thus, *T* is a support tree of *N*^*u*^, and therefore *N*^*u*^ is tree-based.
- If there is a pair of vertices *a* and *b* that were added to *G* when it was constructed to prevent parallel edges between *u* and *v*, we construct a support tree *T* as follows: First, all edges of the cycle in *G* that were already present in *N*^*u*^ are considered. Moreover, except for one fixed pair *a* and *b* that was added to prevent parallel edges, all other such pairs *a′*, *b′* between vertices *u*′ and *v*′ are removed, as we do not have edges {*u′*, *a′*}, {*a′*, *b′*} and {*b′*, *v′*} in *N*^*u*^. (We note that up to permuting the names of *u*′ and *v*′, these edges must be contained in the Hamiltonian cycle; otherwise, *a*′ and *b*′ cannot be covered.) Instead, we add to *T* the corresponding edge {*u′*, *v′*}, which must be contained in *N*^*u*^; otherwise, *a*′ and *b*′ would not have been added during the construction of *G*. Moreover, if the number of leaves of *N*^*u*^ is odd (after a potential pre-processing step), then during the construction of *G*, there may have been another added vertex *a*′′ for the last leaf *x* with attachment point *w*, again to prevent parallel edges between *u′′* and *v*′′. If this is the case, we must have edges {*u*′′, *v*′′}, {*x*, *w*}, {*u*′′, *w*}, and {*w*, *v*′′} in *N*^*u*^. We note that *G* does not contain {*x*, *w*} and {*u*′′, *v*′′}, but {*w*, *a*′′}, {*u*′′, *a*′′}, and {*a*′′, *v*′′}. To cover *a*′′ and *w*, the Hamiltonian cycle must contain the edge {*w*, *a*′′} and either the pair {*u*′′, *a*′′} and {*w*, *v*′′}, or the pair {*v*′′, *a*′′} and {*w*, *u*′′}. In either case, *u*′′ and *v*′′ are covered by the Hamiltonian cycle in *G*, so that one path between them visits only *a*′′ and *b*′′, whereas the other covers all other vertices of *G*. Thus, for *T*, we retain edge {*u*′′, *v*′′} as a replacement for the path containing *a*′′ and *b*′′, and add edges {*x*, *w*} and {*u*′′, *w* } to re-attach leaf *x*. Subsequently, we re-attach all other leaves of *N*^*u*^.Finally, we should handle the fixed pair *a* and *b*. As before, these two vertices can only be covered by the Hamiltonian cycle of *G* if *u* and *v* are connected via one path visiting all vertices of *G* except *u* and *v*, and by one path using only *a* and *b*. However, the existence of *a* and *b* implies that there is an edge {*u*, *v*} in *N*^*u*^. For *T*, we do not consider this edge, that is, we do not translate it from the Hamiltonian cycle of *G* into *N*^*u*^. Thereby, when we delete *a* and *b* (this is required as they are not present in *N*^*u*^), *u* and *v* will be connected via a path visiting all inner vertices of *N*^*u*^, but as the edge {*u*, *v*} is not contained in *T*, *T* is acyclic. Moreover, by construction *T* covers all vertices of *N*^*u*^. As it was created from a Hamiltonian cycle, it is clear that all vertices along this cycle have degree at least 2 in *T*, except for *u* and *v*, which is where we broke the cycle. However, as *u* and *v* are attachment points of leaves, they have degree at least 2 in *T* as well. Thus, in total, all inner vertices of *N*^*u*^ are inner vertices of *T* as well. Thus, *T* is a support tree of *N*^*u*^, and hence *N*^*u*^ is tree-based.

4. We now assume that $G\in \mathcal{LCON}\left({N}^u\right)$ is Hamiltonian and *G* contains two vertices *a* and *b* that were added when two leaf attachment points *u* and *v* were joined in the construction of *G* from *N*^*u*^. As we have seen before, to cover *a* and *b*, the Hamiltonian cycle must contain a path from *u* to *v* visiting only *a* and *b* (and another path from *u* to *v* visiting all other vertices of *G*). Accordingly, the edge {*a*, *b*} must be used. That *N*^*u*^ is tree-based now follows from Part 3 of this theorem.

This completes the proof.

We are now in the position to show that some classes of phylogenetic networks are tree-based using well-known graph theoretical properties.

**Corollary 3** *Let N*^*u*^ *be a proper unrooted phylogenetic network with at least two leaves and such that* $\mathcal{LCUT}\left({N}^u\right)$ *is not Hamiltonian and such that there is a graph G in* $\mathcal{LCON}\left({N}^u\right)$ *which is a 10-tough chordal graph. Then, N*^*u*^ *is tree-based.*

*Proof* According to [8], every 10-tough chordal graph is Hamiltonian. Thus, *G* is Hamiltonian. However, as $\mathcal{LCUT}\left({N}^u\right)$ is not Hamiltonian, the cycle in *G* must use edges that are not contained in *N*^*u*^. Thus, *N*^*u*^ is tree-based by Theorem 5, Part 3. This completes the proof.

**Fig. 12:**
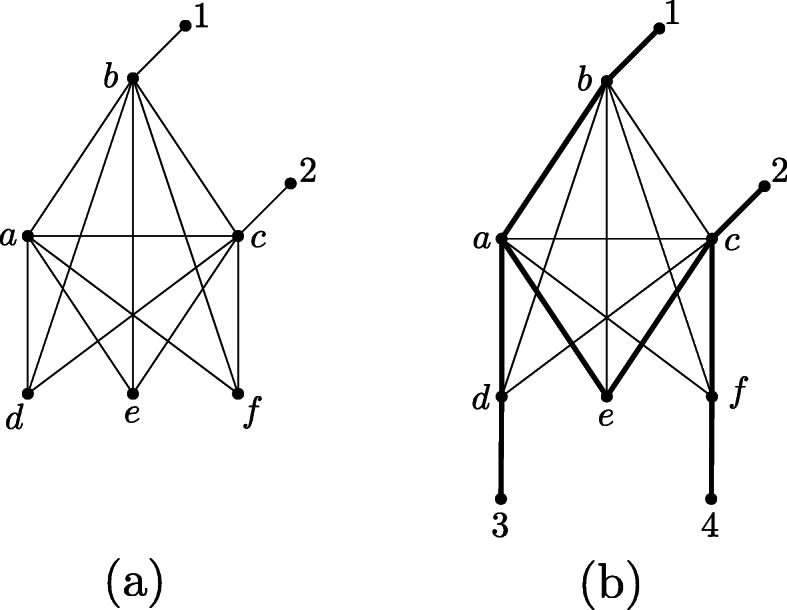
**a** Chordal graph that – considered as an unrooted non-binary phylogenetic network – is not tree-based, because there is no Hamiltonian path between leaves 1 and 2. **b** Attaching at least two more leaves to either d, e or f produces a tree-based network (a support tree is depicted in bold)

We note that even though Corollary 3 implies a connection between chordal graphs and tree-basedness, not all chordal graphs are tree-based. This can be seen in Fig. 12. However, we will now prove that this cannot happen when *N*^*u*^ is binary.

**Theorem 6** *Let N*^*u*^ *be a proper unrooted phylogenetic network with at least two leaves. Then, if N*^*u*^ *is binary and chordal, N*^*u*^ *is edge-based (and thus, by Theorem 3, also tree-based).*

*Proof* Let *N*^*u*^ be a proper unrooted phylogenetic network with at least two leaves, so that *N*^*u*^ is binary and chordal. If *N*^*u*^ is a tree, there is nothing to show because *N*^*u*^ is trivially edge-based and tree-based. Thus, we assume that *N*^*u*^ is not a tree. This implies that *N*^*u*^ must contain at least one non-trivial blob (if it contained only trivial blobs, *N*^*u*^ would be a tree).

By Proposition 1, it now suffices to consider such a non-trivial blob of *N*^*u*^, which we denote by *G*. As *G* is a non-trivial blob, *G* has no cut edges and no leaves; in particular, *G* has only vertices of degree 2 and 3, and as *N*^*u*^ has leaves, the existence of a degree-2 vertex *u* in *G* is ensured. Moreover, *G* is still chordal (as the deletion of leaves does not affect chordality). We now note that in the given chordal graph, every vertex belongs to a triangle by Lemma 9 in Appendix. Therefore, this applies also to *u*; thus, *u* and its neighbors *v* and *w* form a triangle.

**Fig. 13:**
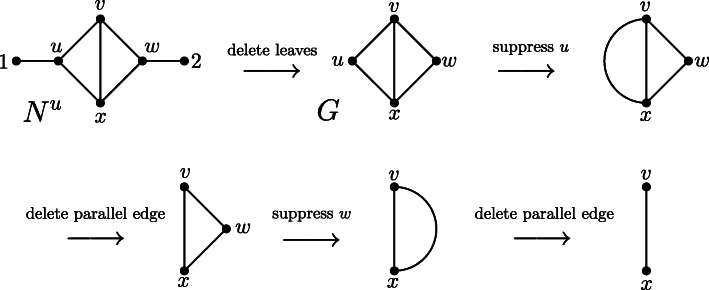
Proper unrooted phylogenetic network N^u^ (consisting of one non-trivial blob and two trivial blobs (leaves)) that is binary and chordal. After deleting its leaves, it can be reduced to a single edge by a sequence of vertex suppression and edge deletion operations. First, we consider the triangle uvx and suppress u. This results in a parallel edge between v and x, which gets deleted. Then, the triangle vwx is considered and w is suppressed. Deleting the resulting parallel edge between v and x leads to a single edge. This implies that N^u^ is edge-based

Accordingly, we have a chordal graph in which all vertices have degree at least 2 and at most 3, and we have one vertex *u* of degree 2, which belongs to a triangle *uvw*. We now repeat the following procedure:

First, we suppress *u*. As *v* and *w* are adjacent (they belong to the triangle *uvw*), we have a parallel edge *e* = {*v*, *w*}. Deleting this parallel edge will strictly decrease the degrees of *v* and *w*. Thus, if the degrees of *v* and *w* were both 2 before the deletion of the parallel edge, we now obtain two new leaves. However, in this case, the edge *e* = {*v*, *w*} is the only remaining edge, and thus *N*^*u*^ is edge-based. If now *v* or *w* has degree 2 after the deletion of the parallel edge, we re-name this vertex as *u*. Again, as the current graph is still chordal (we did not increase the cycle length of any cycle), the new vertex *u* of degree 2 belongs to a triangle, whose suppression yields a parallel edge, and so forth. We can repeat this procedure, as shown in Fig. 13, until only one edge remains. This completes the proof.

**Fig. 14:**
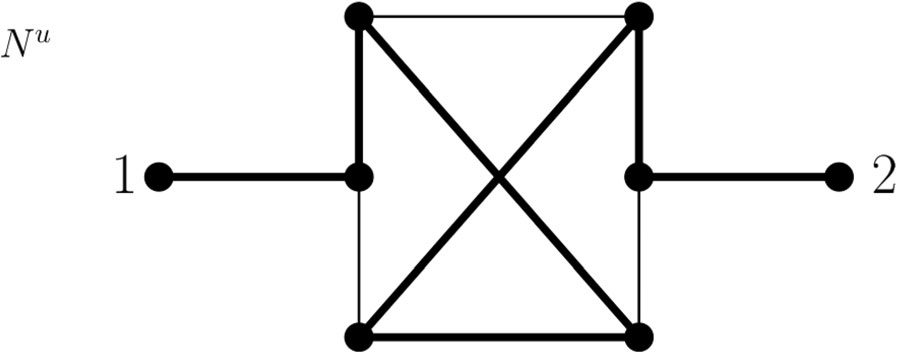
Proper phylogenetic network such that LCUT(N^u^) is a perfect graph. N^u^ is tree-based (the support tree is highlighted in bold), but not edge-based

*Remark 4* A generalization of chordal graphs are the so-called perfect graphs (also known as *Berge* graphs). A perfect graph is a graph *G* such that neither *G* nor its complement $\overline{G}$ contains an odd cycle of length greater than or equal to 5. An interesting question is whether the fact that all binary chordal networks are edge-based (Theorem 6) generalizes to binary perfect networks. If we only consider $\mathcal{LCUT}\left({N}^u\right)$, this is not necessarily the case, as there are networks *N*^*u*^ such that $\mathcal{LCUT}\left({N}^u\right)$ is perfect but not edge-based (Fig. 14).

#### Relationships between different classes of tree-based networks

In the previous sections, we introduced a variety of networks that are necessarily tree-based, ranging from edge-based to $\mathcal{H}$-connected networks. We conclude this section by analyzing the relationships between these classes.

**Fig. 15:**
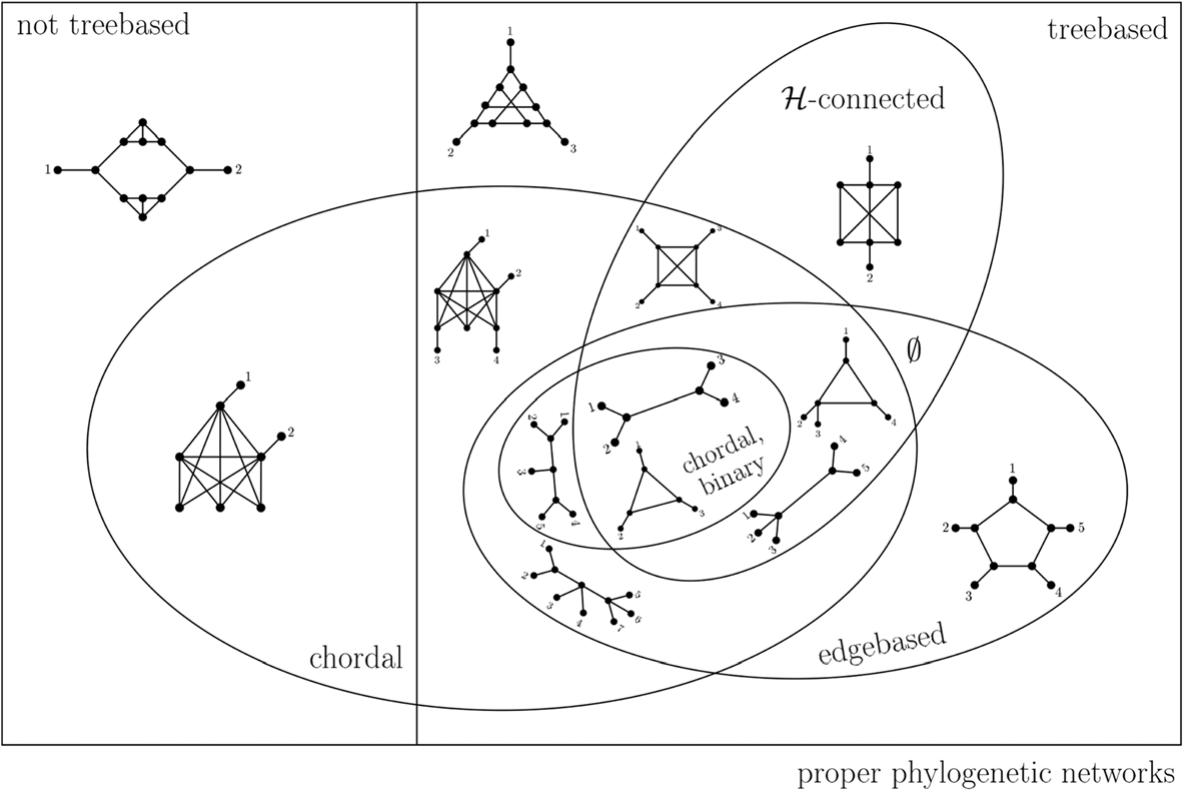
Venn diagram of different classes of proper phylogenetic networks and their connection to tree-basedness

Fig 15 shows a Venn diagram of different classes of proper phylogenetic networks in connection with tree-basedness.

Whenever the intersection of different classes of such networks is non-empty, Fig. 15 contains representative examples. To summarize, we have the following.

- There exist proper phylogenetic networks that are tree-based (Fig. 6 in [5]).
- Not all proper phylogenetic networks are tree-based (Fig. 7 in [5]).
- All proper edge-based phylogenetic networks are tree-based (Theorem 3).
- All proper binary and chordal phylogenetic networks are edge-based and thus tree-based (Theorem 6).
- Proper chordal phylogenetic networks are not necessarily tree-based (Fig. 12).
- Proper $\mathcal{H}$-connected phylogenetic networks are tree-based (Theorem 5, Part 2).

However, we note that the intersection of networks that are edge-based, $\mathcal{H}$-connected, and non-chordal is empty because such networks do not exist. We will explain this subsequently (Remark 5). Moreover, even if the network is chordal, the classes of $\mathcal{H}$-connected and edge-based networks have only a small overlap, as we will show in the following (Theorem 7).

Accordingly, these are indeed highly different types of networks. We will subsequently fully characterize their overlap, that is, we will describe which phylogenetic networks are $\mathcal{H}$-connected and edge-based. In particular, we will show that they are all chordal. We begin with the following theorem.

**Theorem 7** *Let N*^*u*^ *be an edge-based and* $\mathcal{H}$*-connected phylogenetic network. Then,* $\mathcal{LCUT}\left({N}^u\right)$ *contains less than four vertices.*

*Remark 5* This theorem in fact shows that there are no edge-based, $\mathcal{H}$-connected, and non-chordal phylogenetic networks because non-chordal networks require a cycle of length at least 4 (without a chord) and thus at least four vertices in $\mathcal{LCUT}\left({N}^u\right)$.

Before we can prove Theorem 7, two more lemmas are required.

**Lemma 6** *Let N*^*u*^ *be an* $\mathcal{H}$*-connected phylogenetic network such that* $\mathcal{LCUT}\left({N}^u\right)$ *consists of more than just one edge. Then,* $\mathcal{LCUT}\left({N}^u\right)$ *contains no cut vertices and no cut edges.*

*Proof* Let *N*^*u*^ be an $\mathcal{H}$-connected phylogenetic network such that $\mathcal{LCUT}\left({N}^u\right)$ consists of more than one edge. We assume that $\mathcal{LCUT}\left({N}^u\right)$ contains a cut vertex *v*. Then there are at least two more vertices *u* and *w* that become disconnected by the removal of *v*. Thus, the only paths from *u* to *w* in $\mathcal{LCUT}\left({N}^u\right)$ are all via *v*. This implies that there cannot be a Hamiltonian path from *u* to *v* because any sequence of vertices starting at *u* and proceeding through *w* (and possibly other vertices) to *v* would visit *v* at least twice. Thus, if *N*^*u*^ contains cut vertices, *N*^*u*^ is not $\mathcal{H}$-connected, which is a contradiction.

If now $\mathcal{LCUT}\left({N}^u\right)$ contains a cut edge *e* = {*u*, *v*}, this implies that *u* and *v* are cut vertices, leading to a contradiction. This completes the proof.

**Lemma 7** *Let G* = (*V*, *E*) *be a Hamilton-connected graph with at least 4 vertices. Then for all v* ∈ *V, we have deg*(*v*) > 2*.*

*Proof* We first note that in a Hamilton-connected graph, there are clearly no isolated vertices, that is, deg(*v*) > 0 for all *v* ∈ *V*. Moreover, there cannot be any vertices of degree 1 in *G* because, by the same arguments used in the proof of Lemma 6, *G* cannot contain a cut edge (but each edge incident to a leaf would be a cut edge). Thus, deg(*v*) > 1 for all *v* ∈ *V*. Let now *u*, *v*, *w* be in *V* such that deg(*v*) = 2, and *u* and *w* are the two neighbors of *v* in *G*; further, let *x* denote some other vertex in *V*, which must exist as |*V*| ≥ 4. Then, there is no Hamiltonian path from *u* to *w* visting both *v* and *x*. If a path from *u* to *w* starts by visiting *v*, *x* cannot be contained in it unless either *u* or *w* is visited twice. If now a path from *u* to *w* visits *x* before *v*, then *v* can only be reached by visiting either *u* or *w* twice. In both cases, the corresponding path from *u* to *w* is not Hamiltonian and this is a contradiction, as *G* is Hamilton-connected. This completes the proof.

We are now in the position to prove Theorem 7.

*Proof of Theorem 7* We assume toward a contradiction that there exists an $\mathcal{H}$-connected and edge-based phylogenetic network *N*^*u*^ such that $\mathcal{LCUT}\left({N}^u\right)$ contains at least four vertices. As *N*^*u*^ is $\mathcal{H}$-connected, by Lemma 7, $\mathcal{LCUT}\left({N}^u\right)$ contains no vertices of degree at most 2 because, by assumption, it contains at least four vertices. We now consider $\mathcal{LS}\left({N}^u\right)$. When we generate $\mathcal{LS}\left({N}^u\right)$ from $\mathcal{LCUT}\left({N}^u\right)$ (we note that we can proceed from *N*^*u*^ to $\mathcal{LS}\left({N}^u\right)$ via $\mathcal{LCUT}\left({N}^u\right)$ as the order of restriction operations is irrelevant by Theorem 2), there are no degree-2 vertices to suppress. Moreover, there are no parallel edges because if $\mathcal{LCUT}\left({N}^u\right)$ contained parallel edges, so would *N*^*u*^, which contradicts the definition of a phylogenetic network. Additionally, there can be no leaves, as this would imply degree-1 vertices (which cannot exist by Lemma 7). Accordingly, there is no leaf to delete, no degree-2 vertex to suppress, and no parallel edge to delete, that is, $\mathcal{LS}\left({N}^u\right)=\mathcal{LCUT}\left({N}^u\right),$ as there is nothing to shrink. As $\left|V\left(\mathcal{LCUT}\left({N}^u\right)\right)\right|\ge 4$, we have $\left|V\left(\mathcal{LS}\left({N}^u\right)\right)\right|\ge 4$, implying that *N*^*u*^ cannot be edge-based. This is a contradiction. Therefore, the assumption is false and such a network cannot exist. This completes the proof.

We now characterize all cases in which a phylogenetic network is $\mathcal{H}$-connected and edge-based. We will show that the number of networks in this class is quite small. In fact, we can fully characterize their $\mathcal{LCUT}$ graphs.

**Theorem 8** *Let N*^*u*^ *be an* $\mathcal{H}$*-connected and edge-based phylogenetic network. Then, one of the following two cases holds:*

- *N*^*u*^ *is a tree with at most one inner edge,* i.e.*,* $\mathcal{LCUT}\left({N}^u\right)$ *consists of either only one vertex or one edge.*
- *N*^*u*^ *contains precisely one cycle, and this cycle is a triangle, and* $\mathcal{LCUT}\left({N}^u\right)$ *consists only of this triangle.*

*In particular, N*^*u*^ *is chordal.*

*Proof* Let *N*^*u*^ be an $\mathcal{H}$-connected and edge-based phylogenetic network. By Theorem 7, $\mathcal{LCUT}\left({N}^u\right)$ contains at most three vertices. We now distinguish two cases:

- If $\left|V\left(\mathcal{LCUT}\left({N}^u\right)\right)\right|\le 2$, then *N*^*u*^ is clearly a tree (because the vertices of $\mathcal{LCUT}\left({N}^u\right)$ cannot form a cycle) with at most one inner edge (because there is at most one edge in $\mathcal{LCUT}\left({N}^u\right)$ as there are at most two vertices). Therefore, the first case of the theorem holds.
- We now assume that $\left|V\left(\mathcal{LCUT}\left({N}^u\right)\right)\right|=3$. Then, we are clearly not in the first case of the theorem, and we may further assume that the three vertices *u*, *v*, and *w* of $\mathcal{LCUT}\left({N}^u\right)$ do not form a cycle. As $\mathcal{LCUT}\left({N}^u\right)$ is connected, *u*, *v*, and *w* form a path, that is, $\mathcal{LCUT}\left({N}^u\right)$ contains precisely two edges *e*_1_ = {*u*, *v*} and *e*_2_ = {*v*, *w*}. Then, both *e*_1_ and *e*_2_ are cut edges, as their removal would disconnect *u* and *w*. As *N*^*u*^ is $\mathcal{H}$-connected, $\mathcal{LCUT}\left({N}^u\right)$ does not contain any cut edges by Lemma 6, and this is a contradiction. Thus, the three vertices *u*, *v*, and *w* must form a triangle. As there cannot be another vertex in $\mathcal{LCUT}\left({N}^u\right)$, this completes the proof.

By Theorem 8, all $\mathcal{H}$-connected and edge-based phylogenetic networks are chordal, and they have either a single vertex, a single edge, or a triangle as their $\mathcal{LCUT}$ graph. However, the number of networks with these properties is not restricted because an arbitrary number of leaves can be attached to such $\mathcal{LCUT}$ graphs.

## Discussion and conclusions

The primary aim of this study was to link tree-basedness of phylogenetic networks to classical graph theory. More precisely, we established links between tree-basedness and the theory of Hamiltonian or Hamilton connected graphs, as well as between tree-basedness and the family of GSP graphs.

The close links of tree-based networks and Hamiltonian or Hamilton connected graphs provide sufficient criteria whereby a network may be tree-based; however, none of these criteria is necessary. It is conceivable that future research will establish even more links between Hamiltonicity of graphs and tree-basedness of phylogenetic networks. Furthermore, as an increasing number of classes of graphs are being discovered to be Hamilton connected [16, 17], an increasing number of known graphs are expected to lead to tree-based networks.

However, none of these links to Hamiltonicity leads to network classes for which tree-basedness can be efficiently verified, as the previously mentioned graph theoretical counterparts of tree-basedness (e.g., testing if a graph is Hamiltonian) are known to be NP-complete [12].

Nevertheless, we introduced a class of networks that are necessarily tree-based, namely, the class of edge-based networks. Interestingly, these networks are closely related to another important concept in classical graph theory, namely, the class of GSP graphs. In the present study, we showed that the links between tree-basedness, edge-basedness and GSP graphs lead to a sufficient criterion for tree-basedness that can be verified in linear time. In this regard, edge-based phylogenetic networks form a class of tree-based networks that can easily be found. For example, we showed that all unrooted, binary, chordal phylogenetic networks are edge-based. As mentioned in Remark 4, an interesting question is whether this generalizes to other classes of proper phylogenetic networks, for example, perfect binary ones. It would also be of interest to analyze whether edge-based networks frequently occur in practice, that is, when phylogenetic networks are constructed from biological data. As research on reconstructing phylogenetic networks from data is still at its beginning, this is difficult to predict. However, it is conceivable that edge-based networks will be of practical relevance in the future.

We concluded our study by analyzing the relationships between the classes of tree-based networks summarized in Fig. 15. It is expected that future research will characterize more classes of tree-based networks, enhancing our results.

## List of important definitions

**Definition** (Unrooted phylogenetic network):

Let *X* denote a finite set with |*X*| ≥ 1. An unrooted phylogenetic network *N*^*u*^ (on *X*) is a connected, simple graph *G* = (*V*, *E*) with *X* ⊆ *V* and no vertices of degree 2, where the set of degree-1 vertices (referred to as the leaves or taxa of the network) is bijectively labeled by *X*. Such an unrooted network is called unrooted binary if every inner vertex *u* ∈ *V* ∖ *X* has degree 3. It is called a phylogenetic tree if the underlying graph structure is a tree.

**Definition** (Tree-based phylogenetic network)

A phylogenetic network *N*^*u*^ = (*V*, *E*) on *X* is called tree-based if there is a spanning tree *T* = (*V*, *E*^′^) in *N*^*u*^ (with *E*′ ⊆ *E*) whose leaf set is equal to *X*. This spanning tree is then called a support tree for *N*^*u*^. Moreover, the tree *T*′ that can be obtained from *T* by suppressing potential degree-2 vertices is called a base tree for *N*^*u*^.

**Definition** (GSP graph (adapted from [11]))

1. The graph *K*_2_ consisting of two vertices *u* and *v* (called terminals) and a single edge {*u*, *v*} is a primitive GSP graph.
2. If *G*_1_ and *G*_2_ are two GSP graphs with terminals *u*_1_, *v*_1_ and *u*_2_, *v*_2_, respectively, then the graph obtained by any of the following three operations is a GSP graph:

- Series composition of *G*_1_ and *G*_2_: identifying *v*_1_ with *u*_2_ and specifying *u*_1_ and *v*_2_ as the terminals of the resulting graph.
- Parallel composition of *G*_1_ and *G*_2_: identifying *u*_1_ with *u*_2_ and *v*_1_ with *v*_2_, and specifying *u*_1_ and *v*_1_ as the terminals of the resulting graph.
- Generalized-series composition of *G*_1_ and *G*_2_: identifying *v*_1_ with *u*_2_ and specifying *u*_2_ and *v*_2_ as the terminals of the resulting graph.

**Definition** (SP graph (adapted from [11]))

1. The graph *K*_2_ consisting of two vertices *u* and *v* (called terminals) and a single edge {*u*, *v*} is a primitive SP graph.
2. If *G*_1_ and *G*_2_ are two SP graphs with terminals *u*_1_, *v*_1_ and *u*_2_, *v*_2_, respectively, then the graph obtained by any of the following two operations is an SP graph:

- Series composition of *G*_1_ and *G*_2_: identifying *v*_1_ with *u*_2_ and specifying *u*_1_ and *v*_2_ as the terminals of the resulting graph.
- Parallel composition of *G*_1_ and *G*_2_: identifying *u*_1_ with *u*_2_ and *v*_1_ with *v*_2_, and specifying *u*_1_ and *v*_1_ as the terminals of the resulting graph.

**Definition** (Leaf cut graph)

Let *N*^*u*^ be a phylogenetic network on taxon set *X* with |*V*(*N*^*u*^)| ≥ 2 and |*X*| ≥ 2. We call the simple graph *G* resulting from deleting all leaves labeled by *X* from *V*(*N*^*u*^) and their incident edges the leaf cut graph of *N*^*u*^ and denote it by $\mathcal{LCUT}\left({N}^u\right)$.

**Definition** ($\mathcal{H}$-connected network)

Let *N*^*u*^ be a proper phylogenetic network such that $\mathcal{LCUT}\left({N}^u\right)$ is Hamilton connected. Then, *N*^*u*^ is called an $\mathcal{H}$*-*connected network.

**Definition** (Leaf shrink graph)

Let *G* be a simple graph with |*V*(*G*)| ≥ 2 and |*V*_*L*_(*G*)| ≥ 2. We call the simple graph resulting from Algorithm 1 the leaf shrink graph of *G* and denote it by $\mathcal{LS}(G)$.

**Definition** (Edge-based graph/network)

Let *G* be a connected graph with |*V*(*G*)| ≥ 2 and |*V*_*L*_(*G*)| ≥ 2. If the leaf shrink graph $\mathcal{LS}(G)$ of *G* is a single edge, *G* is called edge-based. Else, *G* is called non-edge-based. If *G* = *N*^*u*^ is a proper phylogenetic network with |*V*(*N*^*u*^)| ≥ 2 and |*X*| ≥ 2 and $\mathcal{LS}\left({N}^u\right)$ is a single edge, we call *N*^*u*^ an edge-based network. Else, *N*^*u*^ is called non-edge-based.

**Definition** (Set of leaf connecting graphs)

Let *N*^*u*^ be a phylogenetic network on *X* (with |*X*| ≥ 2) that is not a tree. We call the set of simple graphs resulting from the leaf connecting procedure (described on page 5) the set of leaf connecting graphs of *N*^*u*^ and denote it by $\mathcal{LCON}\left({N}^u\right)$.

## Figures and Tables

**Fig. 16:**
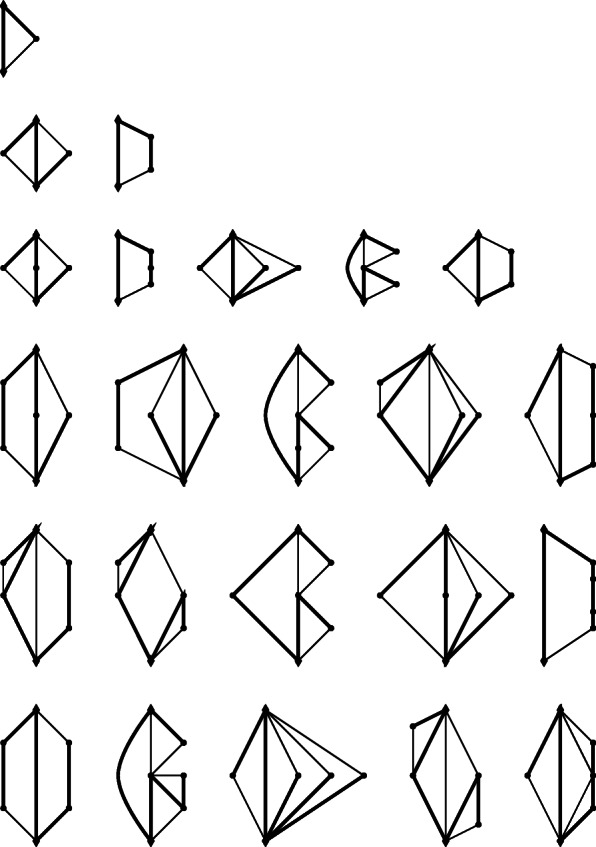
Catalog of all simple and biconnected SP graphs on *n* ≤ 6 vertices (diamond vertices represent one possible pair of terminals, respectively) and a valid spanning tree (depicted in bold)

**Fig. 17:**
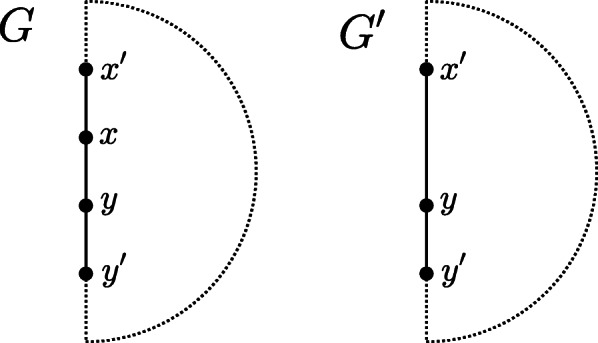
Case 1 in the proof of Lemma 5. The dotted lines depict some path between *x*′ and *y*′ that must exist since *G* is biconnected

**Fig. 18:**
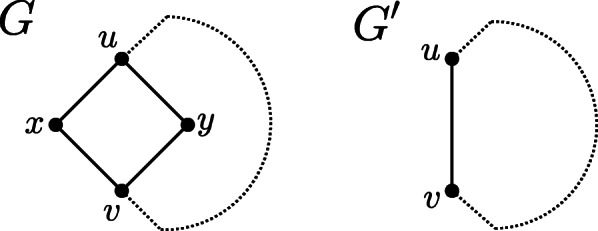
Case 2 in the proof of Lemma 5 for *n* ≥ 7. The dotted line depicts some path between *u* and *v* (possibly consisting of a single edge) that must exist since *G* is biconnected and *n* ≥ 7

**Fig. 19:**
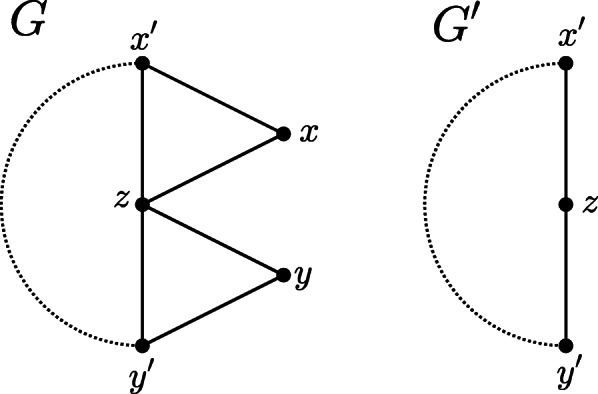
Case 3 in the proof of Lemma 5. The dotted line depicts some path between *x*′ and *y*′ which must exist since *G* is biconnected and *n* ≥ 7

**Fig. 20:**
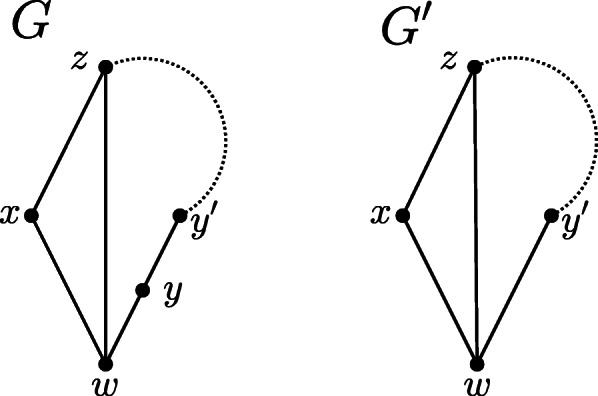
Case 4 in the proof of Lemma 5. The dotted line depicts some path between *z* and *y*′ which must exist since *G* is biconnected and *n* ≥ 7

**Fig. 21:**
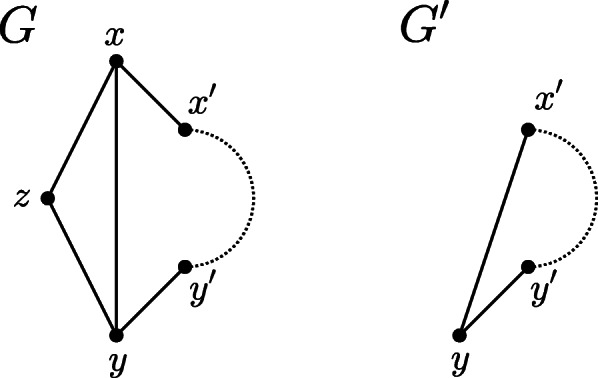
Case 5 in the proof of Lemma 5 for *n* ≥ 7. The dotted line depicts some path between *x* and *y*′ which must exist since *G* is biconnected and *n* ≥ 7

**Fig. 22:**
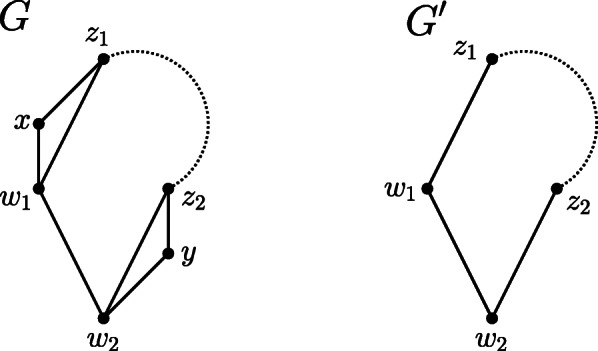
Case 6 in the proof of Lemma 5 for *n* ≥ 7. The dotted line depicts some path between *z*_1_ and *z*_2_ which must exist since *n* ≥ 7 and *G* is biconnected

**Fig. 23:**
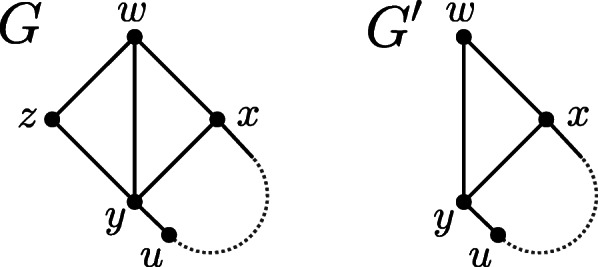
Case 7 in the proof of Lemma 5. The dotted line depicts some path between *u* and *x* which must exist since *n* ≥ 7 and *G* is biconnected

**Fig. 24:**
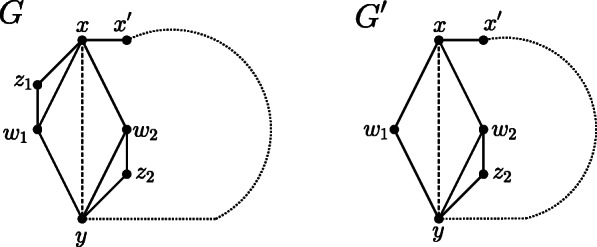
Case 8 in the proof of Lemma 5 for *n* ≥ 7. The dashed edge {*x*, *y*} may be present or not and the dotted line depicts some path between *x*′ and *y*, which must exist since *G* is biconnected and *n* ≥ 7

**Fig. 25:**
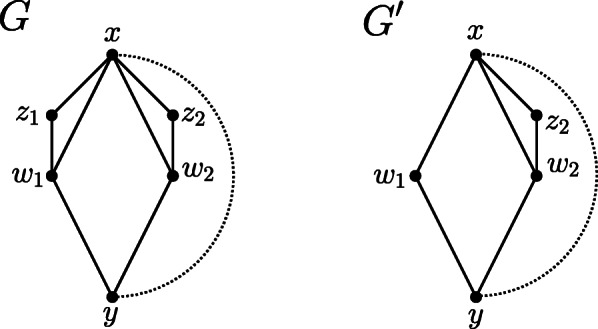
Case 9 in the proof of Lemma 5. The dotted line depicts some path between *x* and *y* which must exist as *n* ≥ 7 (since *G* is biconnected)

**Fig. 26:**
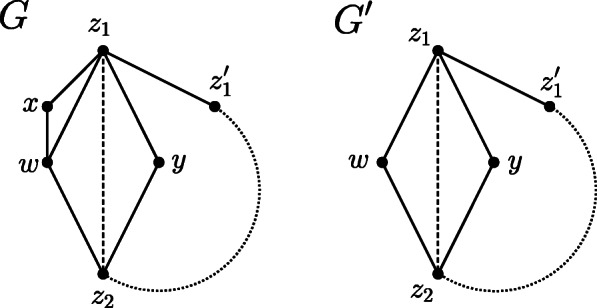
Case 10 in the proof of Lemma 5 for *n* ≥ 7. The dashed edge {*z*_1_, *z*_2_} may either be present or absent and the dotted line depicts some path between *z*_1_′ and *z*_2_ which must exist since *G* is biconnected

## Data Availability

Not applicable.

## Appendix

**Lemma 3** *Let G be a connected graph with vertex set V(G) and edge set E(G). Let G′ result from G by deleting one loop. Then, a graph H (with H ≠ G) is a restricted topological subgraph of G if and only if H is a restricted topological subgraph of G′.*

*Proof* By Lemma 2, if *H* is a restricted topological subgraph of *G*′, then it is also a restricted topological subgraph of *G*, and thus this direction is clear.

We now assume that there is a graph *G* such that *H* is a restricted topological subgraph of *G*, but if we delete one loop of *G* to obtain *G*′, *H* no longer is a restricted topological subgraph. If such graphs exist, we may consider a minimal one in terms of the number of edges. Thus, we assume that *G* is minimal with this property, that is, for all graphs with fewer edges, we know that if *H* is a restricted topological subgraph, this property still holds after the deletion of a loop.

As *G* has *H* as a restricted topological subgraph, there is a sequence of the restriction operations that convert *G* into *H*. However, there is also a loop {*u*, *u*} whose deletion converts *G* into *G*′. Thus, the first operation to convert *G* into *H* cannot be the deletion of this loop. Accordingly, the first step is either the deletion of a leaf (together with its incident edge), the suppression of a degree-2 vertex (which ‘melts’ two edges into one), the deletion of one copy of a parallel edge, or the deletion of some other loop. In all cases, we obtain a graph *G*′′ containing fewer edges than *G* and having *H* as a restricted topological subgraph, as it is on the path from *G* to *H*. However, as *G* is minimal with the property that the deletion of a loop can cause a loss of *H* as a restricted topological subgraph, we can delete the loop {*u*, *u*} from *G*′′ to obtain $\overset{\sim }{G}$, which again has *H* as a restricted topological subgraph. By Lemma 2, we can undo the first step from *G* to *G*′′, that is, we can re-add the deleted leaf, degree-2 vertex, parallel edge or loop (we note that this implies we convert $\overset{\sim }{G}$ into *G*′), without losing the property that *H* is a restricted topological subgraph. Thus, *H* is a restricted topological subgraph of *G*′, which contradicts our assumption. Therefore, such graphs cannot exist, implying that the question whether *H* is a restricted topological subgraph of a graph *G* cannot depend on the loops of *G*. This completes the proof.

**Lemma 4** *Let G be a connected graph with vertex set V(G) and edge set E(G). Let G′ result from G by deleting one copy of a parallel edge. Then, a graph G (with H ≠ G) is a restricted topological subgraph of G if and only if H is a restricted topological subgraph of G*′.

*Proof* By Lemma 2, if *H* is a restricted topological subgraph of *G*′, then it is also a restricted topological subgraph of *G*; thus, this direction is clear.

We now assume that there is a graph *G* such that *H* is a restricted topological subgraph of *G*, but if we delete a copy of a parallel edge of *G* to obtain *G*′, *H* no longer is a restricted topological subgraph. If such graphs exist, we may consider a minimal one in terms of the number of edges. Thus, we assume that G is minimal with this property, that is, for all graphs with fewer edges we know that if *H* is a restricted topological subgraph, this property still holds after the deletion of a parallel edge.

As *G* has *H* as a restricted topological subgraph, there is a sequence of restriction operations that convert *G* into *H*. However, there is also an edge *e* for which multiple copies exist, such that the deletion of *e* converts *G* into *G*′. Accordingly, the first operation to convert *G* into *H* cannot be the deletion of *e*. Thus, the first step is either the deletion of a leaf (together with its incident edge), the suppression of a degree-2 vertex (which ‘melts’ two edges into one), the deletion of one copy of a parallel edge other than *e*, or the deletion of a loop. In all cases, we obtain a graph *G*′′ containing fewer edges than *G* and having *H* as a restricted topological subgraph, as it is on the path from *G* to *H*. However, as *G* is minimal with the property that the deletion of a parallel edge can cause a loss of *H* as a restricted topological subgraph, if *e* is contained in *G*′′, we can delete one copy of *e* from *G*′′ to obtain $\overset{\sim }{G}$, which again has *H* as a restricted topological subgraph. By Lemma 2, we can now undo the first step from *G* to *G*′′, that is, we can re-add the deleted leaf, degree-2 vertex, parallel edge, or loop (we note that this implies that we convert $\overset{\sim }{G}$ into *G*′) to $\overset{\sim }{G}$, without losing the property that *H* is a restricted topological subgraph. Thus, *H* is a restricted topological subgraph of *G*′, which contradicts our assumption.

If now *G*′′ does not contain *e*, then one concludes that *e* disappeared in the transformation of *G* into *G*′′ by one of the other operations. We note that a leaf deletion only affects a degree-1 vertex and its incident edge, which thus cannot be a parallel edge (otherwise, the vertex would have degree at least 2). Moreover, the deletion of a loop (even if it was parallel, that is, even if it existed multiple times) would not cause the disappearance of an edge *e* that is present multiple times in *G*. Neither would the deletion of another parallel edge unrelated to *e*. Thus, *e* may disappear in the first step only if there are precisely two copies of *e* = {*u*, *v*} that lead to a vertex *v* that is incident only to these two edges *e*, that is, deg(*v*) = 2. Then, the suppression of *v* would lead to a loop {*u*, *u*}, and indeed no copy of *e* would be present in *G*′′. However, in this case, by Lemma 3, we can delete loop {*u*, *u*} to obtain *G*′′′, and *G*′′′ still has *H* as a restricted topological subgraph. As above, we can now undo the first step (from *G* to *G*′′) in *G*′′′ by Lemma 2. This leads to a graph $\overset{\sim }{G}$ that still has *H* as a restricted topological subgraph. Again by Lemma 2, we can then add vertex *v* and connect it to vertex *u* with one new edge *e* = {*u*, *v*}. This is equivalent to introducing a new leaf, thus preserving *H* as a restricted topological subgraph. However, the resulting graph is *G*′, which cannot have *H* as a restricted topological subgraph by assumption. Therefore, this is a contradiction.

Accordingly, in both cases, we arrive at a contradiction, and therefore such graphs cannot exist. Hence, the question whether *H* is a restricted topological subgraph of a graph *G* cannot depend on copies of multiple edges. This completes the proof.

**Lemma 5** *Let G = (V, E) be a simple and biconnected SP graph with at least three vertices. Then, there exists a spanning tree T in G whose leaves correspond to degree-2 vertices of G. In particular, no vertex v ∈ V (G) with deg(v) > 2 is a leaf in T*.

We note that such a spanning tree is called a *valid* spanning tree (Remark 3). To prove Lemma 5, we require the following lemma by [22], in which *N*(*v*) denotes the neighborhood of a vertex *v* in *G*, that is, the set of vertices adjacent to *v*.

**Lemma 8** (adapted from [22])

*Let G* = (*V*, *E*) *be a simple and biconnected SP graph with* |*V*| ≥ 5*. Then one of the following conditions holds:*

1. *G has two adjacent degree-2 vertices x and y;*
2. *G has two different degree-2 vertices x and y and N*(*x*) = *N*(*y*)*;*
3. *G has a degree-4 vertex z adjacent to two degree-2 vertices x and y such that N*(*z*) \ {*x*, *y*} = {*N*(*x*) ∪ *N*(*y*)} \ {*z*}*;*
4. *G has a degree-3 vertex w with N*(*w*) = {*x*, *y*, *z*} *such that both x and y are degree-2 vertices, N*(*x*) = {*z*, *w*} *and edge* {*y*, *z*} ∉ *E;*
5. *G has two adjacent degree-3 vertices x and y such that N*(*x*) ∩ *N*(*y*) = {*z*} *and N*(*z*) = {*x*, *y*}*;*
6. *G has two adjacent degree-3 vertices w*_1_ *and w*_2_ *such that N*(*w*_1_) = {*x*, *z*_1_, *w*_2_}*, N*(*w*_2_) = {*y*, *z*_2_, *w*_1_}*, N*(*x*) = {*z*_1_, *w*_1_} *and N*(*y*) = {*z*_2_, *w*_2_}*;*
7. *G has a degree-3 vertex w with N*(*w*) = {*x*, *y*, *z*} *such that N*(*z*) = {*w*, *y*} *and edge* {*x*, *y*} ∈ *E;*
8. *G has two non-adjacent degree-3 vertices w*_1_ *and w*_2_ *such that N*(*w*_1_) = {*x*, *y*, *z*_1_}*, N*(*w*_2_) = {*x*, *y*, *z*_2_}*, N*(*z*_1_) = {*x*, *w*_1_} *and N*(*z*_2_) = {*y*, *w*_2_}*;*
9. *G has two non-adjacent degree-3 vertices w*_1_ *and w*_2_ *such that N*(*w*_1_) = {*x*, *y*, *z*_1_}*, N*(*w*_2_) = {*x*, *y*, *z*_2_}*, N*(*z*_1_) = {*x*, *w*_1_} *and N*(*z*_2_) = {*x*, *w*_2_};
10. *G has a degree-3 vertex w with N*(*w*) = {*x*, *z*_1_, *z*_2_} *such that there is a degree-2 vertex y* ∈ *N*(*z*_1_) ∩ *N*(*z*_2_) *and N*(*x*) = {*z*_1_, *w*}*.*

Based on this we can now prove Lemma 5.

*Proof of Lemma 5* We use induction on the number *n* ∶  =  ∣ *V*∣ of vertices of *G*. For *n* = 3, …, 6, we generated a catalog of all simple and biconnected SP graphs with *n* vertices as follows: We retrieved all simple and connected graphs with *n* = 3, …, 6 vertices from the “House of graphs” database [21] and filtered them for biconnected SP graphs using the computer algebra system Mathematica [23]. First, each downloaded graph *G* was checked for biconnectedness using the Mathematica function KVertexConnectedGraphQ[*G*, 2]. For each of the remaining graphs, it was then checked whether a reduction to *K*_2_ via the series reduction rules (page 3) was possible. If not, the graph was discarded. The remaining simple and biconnected SP graphs are shown in Fig. 16. We exhaustively analyzed all these graphs to show that in all cases, there exists a valid spanning tree having only degree-2 vertices of the respective SP graph as leaves (which is also shown in Fig. 16). This completes the base case of the induction.

We now assume that the statement holds for all simple and biconnected SP graphs with up to *n* − 1 vertices and let *G* = (*V*, *E*) be a simple and biconnected SP graph with *n* ≥ 7 vertices.

By Lemma 8, we can distinguish ten cases:

1. *G* has two adjacent degree-2 vertices *x* and *y* (as shown in Fig. 17): Let *x*′ ≠ *y* be the second vertex adjacent to *x* and let *y*′ ≠ *x* be the second vertex adjacent to *y*. We note that as *n* ≥ 7, we cannot have *y*′ = *x*′, because in this case, *x*′ would be a cut vertex, contradicting the fact that *G* is biconnected. We now construct a simple and biconnected SP graph *G*′ with *n* − 1 vertices from *G* by suppressing vertex *x* (Fig. 17). By the inductive hypothesis (as *G*′ is a simple and biconnected SP graph on 6 ≤ *n* − 1 < *n* vertices), there exists a valid spanning tree *T*′ in *G*′. We can now obtain a valid spanning tree *T* for *G* as follows:
  - If edge {*x*′, *y*} ∈ *E*(*T*^′^) (we note that {*x*′, *y*} ∈ *E*(*G*′) \ *E*(*G*)), we replace this edge by {*x*^′^, *x*} and {*x*, *y*} to obtain *T*. In this case, *x* is not a leaf of *T*.
  - If edge {*x*′, *y*} ∉ *E*(*T*′), we add either {*x*′, *x*} or {*y*, *x*} to *T*′ to obtain *T*. This implies that *x* is a leaf in *T*, but as *x* was a degree-2 vertex in *G*, this is valid.

In both cases, *T* is a valid spanning tree for *G*.

2. *G* has two different degree-2 vertices *x* and *y* and *N*(*x*) = *N*(*y*) (as shown in Fig. 18): Let *N*(*x*) = *N*(*y*) = {*u*, *v*}. We now construct a simple and biconnected SP graph *G*′ with *n* − 2 vertices from *G* by suppressing vertices *x* and *y*, and deleting all but one copy of the resulting parallel edge {*u*, *v*} (Fig. 18).

As *G*′ is a simple and biconnected SP graph with 5 ≤ *n* − 2 < *n* vertices, by the inductive hypothesis, *G*′ has a valid spanning tree *T*′. We note that *u* and *v* are potentially leaves in *T*′ (as they are potentially degree-2 vertices in *G*′). We now distinguish two cases:

- If edge {*u*, *v*} ∉ *E(T')*, we can for example add edges {*u*, *x*} and *{v,y}* (or *{u,y}* and *{v,x}*) to *T'* to obtain a valid spanning tree *T* for *G*. This ensures that *u* and *v* are interior vertices of *T*, whereas *x* and *y* are leaves (this is allowed because they are degree-2 vertices in *G*).
- If edge {*u*, *v*} *∈ E(T')*, note that at most one of *u* and *v* can be a leaf in *T'* (otherwise, *T'* would not be connected).
  - If *u* is a leaf in *T'*, we can replace edge *{u, v}* by *{u, y}* and *{y, v}*, and add edge *{u, x}* to *T'* to obtain a valid spanning tree *T'* for *G*. This ensures that *u* is not a leaf in *T'*.
  - If *v* is a leaf in *T'*, we can, for example, again replace edge *{u, v}* by *{u, y}* and *{y, v}*, and add edge *{v, x}* to *T'* to obtain a valid spanning tree *T* for *G*. This ensures that *v* is not a leaf in *T*.
  - Finally, if neither *u* nor *v* is a leaf in *T'*, we can, for example, replace edge *{u, v}* by *{u, y}* and *{y, v}*, and add edge *{u, x}* (or *{v, x}*) to *T'* to obtain a valid spanning tree *T* for *G*.

3. *G* has a degree-4 vertex *z* adjacent to two degree-2 vertices *x* and *y* such that *N*(*z*) \ {*x*, *y*} = {*N*(*x*) ∪ *N*(*y*)} \ {*z*} (as shown in Fig. 19): Let *N*(*z*) \ {*x*, *y*} = {*N*(*x*) ∪ *N*(*y*)} \ {*z*} = {*x*′, *y*′}, where *x*′ ∈ *N*(*x*) \ {*z*} and *y*^′^ ∈ *N*(*y*) \ {*z*}. We now construct a simple and biconnected SP graph *G*′ with *n* − 2 vertices from *G* by suppressing vertices *x* and *y* and deleting all but one copy of the resulting parallel edges (Fig. 19). We note that *z* is a degree-2 vertex in *G*′, and *x*′ and *y*′ may also be of degree 2 in *G*′. By the inductive hypothesis (as *G*′ is a simple and biconnected SP graph with 5 ≤ *n* − 2 < *n* vertices), there exists a valid spanning tree *T*′ for *G*′. As *z*, *x*′, and *y*′ are potentially degree-2 vertices in *G*′, they are potentially leaves in *T*′. However, they cannot simultaneously be leaves because *T*′ would not be connected. Thus, we distinguish different cases:

- *x*′ and *y*′ are leaves in *T*′. This cannot happen because *T*′ would not be connected.
- *z* and *y*′ are leaves in *T*′. In this case, we add the edges {*z*, *x*} and {*y*′, *y*} to *T*′ to obtain a valid spanning tree *T* for *G* (in which *z* and *y*′ are internal vertices and *x* and *y* are leaves).
- *x*′ and *z* are leaves in *T*′. In this case, we add the edges {*x*′, *x*} and {*z*, *y*} to *T*′ and obtain a valid spanning tree *T* for *G* (in which *z* and *x*′ are internal vertices, and *x* and *y* are leaves).
- *x*′ is a leaf in *T*′. In this case, we add the edges {*x*′, *x*} and either {*z*, *y*} or {*y*′, *y*} to *T*′ and obtain a valid spanning tree *T* for *G* (in which *x*′ is an internal vertex, and *x* and *y* are leaves).
- *y*′ is a leaf in *T*′. In this case, we add the edges {*y*′, *y*} and either {*z*, *x*} or {*x*′, *x*} to *T*′ and obtain a valid spanning tree *T* for *G* (in which *y*′ is an internal vertex, and *x* and *y* are leaves).
- *z* is a leaf in *T*′. In this case, we add the edges {*z*, *x*} and {*y*′, *y*} (or {*z*, *x*} and {*z*, *y*}, or {*z*, *y*} and {*x*′, *x*}) to *T*′ and obtain a valid spanning tree *T* for *G* (in which *z* is an internal vertex, and *x* and *y* are leaves).
- Neither *x*′, *y*′, nor *z* is a leaf in *T*′. In this case, we can, for example, add the edges {*x*′, *x*} and {*y*′, *y*} to *T*′ and obtain a valid spanning tree *T* for *G*.

4. *G* has a degree-3 vertex *w* with *N*(*w*) = {*x*, *y*, *z*} such that both *x* and *y* are degree-2 vertices, *N*(*x*) = {*z*, *w*}, and edge {*y*, *z*} ∉ *E* (as shown in Fig. 20): Let *y*′ ≠ *w* be the second vertex adjacent to *y*. We cannot have *y*′ = *z* because {*y*, *z*} ∉ *E*. We now construct a simple and biconnected SP graph *G*′ with *n* − 1 vertices from *G* by suppressing vertex *y* (Fig. 20). By the inductive hypothesis (as *G*′ is a simple and biconnected SP graph with 6 ≤ *n* − 1 < *n* vertices), *G*′ has a valid spanning tree *T*′, and we can obtain a valid spanning tree *T* for *G* from *T*^′^ as follows:

- If edge {*w*, *y*′} ∈ *E*(*T*′), we replace this edge by the edges {*w*, *y*} and {*y*, *y*′} to obtain *T*.
- If edge {*w*, *y*′} ∉ *E*(*T*′), we add either {*w*, *y*} or {*y*′, *y*} to *T*′ to obtain *T*, that is, we add *y* as a leaf to *T* (this is allowed because *y* has degree 2 in *G*).

5. *G* has two adjacent degree-3 vertices *x* and *y* such that *N*(*x*) ∩ *N*(*y*) = {*z*} and *N*(*z*) = {*x*, *y*} (as shown in Fig. 21): Let *x*′ be the vertex in *N*(*x*) \ {*y*, *z*}, and let *y*′ be the vertex in *N*(*y*) \ {*x*, *z*}. We now construct a simple and biconnected SP graph *G*′ with *n* − 2 vertices from *G* as follows (Fig. 21):

- Suppress the degree-2 vertex *z*.
- Delete one copy of the resulting parallel edge {*x*, *y*}.
- Suppress the resulting degree-2 vertex *x*.

As *G*′ is a simple and biconnected SP graph with 5 ≤ *n* − 2 < *n* vertices, by the inductive hypothesis, *G*′ has a valid spanning tree *T*′. We note that as *y* is a degree-2 vertex in *G*′, it may be a leaf in *T*′. We now construct a valid spanning tree *T* for *G* from *T*′ by distinguishing two cases:

- If edge {*x*′, *y*} ∈ *E*(*T*′) (we note that {*x*′, *y*} ∈ *E*(*G*′) \ *E*(*G*)), we replace edge {*x*′, *y*} by edges {*x*′, *x*} and {*x*, *y*}, and add edge {*y*, *z*} to obtain *T*. This ensures that the degree-3 vertices *x* and *y* of *G* are not leaves in *T*, and thus *T* is a valid spanning tree for *G* (we note that *z* is a leaf in *T*, but as deg(*z*) = 2 in *G*, this is valid).
- If edge {*x*′, *y*} ∉ *E*(*T*′), we add the edges {*y*, *x*} and {*x*, *z*} to *T*′ to obtain *T*. Again, *z* is a leaf in *T*, but *x* and *y* are not, and thus *T* is a valid spanning tree for *G*.

6. *G* has two adjacent degree-3 vertices *w*_1_ and *w*_2_ such that *N*(*w*_1_) = {*x*, *z*_1_, *w*_2_}, *N*(*w*_2_) = {*y*, *z*_2_, *w*_1_}, *N*(*x*) = {*z*_1_, *w*_1_}, and *N*(*y*) = {*z*_2_, *w*_2_} (as shown in Fig. 22):

We note that as *n* ≥ 7, *z*_1_ and *z*_2_ are distinct; otherwise, *z*_1_ = *z*_2_ would be a cut vertex, contradicting the fact that *G* is biconnected. We now construct a simple and biconnected SP graph *G*′ with *n* − 2 vertices from *G* as follows (Fig. 22):

- Suppress the degree-2 vertex *x* and delete one copy of the resulting parallel edge {*z*_1_, *w*_1_}.
- Suppress the degree-2 vertex *y* and delete one copy of the resulting parallel edge {*z*_2_, *w*_2_}.

We note that *w*_1_ and *w*_2_ are degree-2 vertices in *G*′ and *z*_1_ and *z*_2_ may be of degree 2 in *G*′ as well.

By the inductive hypothesis (as *G*′ is a simple and biconnected SP graph with 5 ≤ *n* − 2 < *n* vertices), *G*′ has a valid spanning tree *T*′, in which *w*_1_, *w*_2_, *z*_1_, and *z*_2_ are potentially leaves. However, at most two of them can simultaneously be leaves in *T*′; otherwise, *T*′ would be disconnected. We now construct a valid spanning tree *T* for *G* from *T*′ by distinguishing the following cases:

- If *w*_1_, *w*_2_, *z*_1_, and *z*_2_ are internal vertices of *T*′, we can, for example, add the edges {*w*_1_, *x*} and {*w*_2_, *y*} to *T*′ and obtain a valid spanning tree *T* for *G*.
- If *w*_1_ is a leaf in *T*′ (and *w*_2_, *z*_1_ and *z*_2_ are internal vertices in *T*′), we add the edge {*w*_1_, *x*} and either {*w*_2_, *y*} or {*z*_2_, *y*} to *T*′ and obtain a valid spanning tree *T* for *G*.
- If *w*_2_ is a leaf in *T*′ (and *w*_1_, *z*_1_, and *z*_2_ are internal vertices in *T*′), we add the edge {*w*_2_, *y*} and either {*w*_1_, *x*} or {*z*_1_, *x*} to *T*′ and obtain a valid spanning tree *T* for *G*.
- If *z*_1_ is a leaf in *T*′ (and *w*_1_, *w*_2_, and *z*_2_ are internal vertices in *T*′), we add the edge {*z*_1_, *x*} and either {*w*_2_, *y*} or {*z*_2_, *y*} to *T*′ and obtain a valid spanning tree *T* for *G*.
- If *z*_2_ is a leaf in *T*′ (and *w*_1_, *w*_2_ and *z*_1_ are internal vertices in *T*′), we add the edge {*z*_2_, *y*} and either one of the edges {*w*_1_, *x*} or {*z*_1_, *x*} to *T*′ and obtain a valid spanning tree *T* for *G*.
- *z*_1_ and *z*_2_ are leaves in *T'* (and *w*_1_ and *w*_2_ are internal vertices in *T'*). This case cannot happen because *T'* would not be connected.
- If *w*_1_ and *w*_2_ are leaves in *T*′ (and *z*_1_ and z_2_ are internal vertices in *T*′), we add the edges {*w*_1_, *x*} and {*w*_2_, y} to *T*′ and obtain a valid spanning tree *T* for *G*.
- If *w*_1_ and *z*_1_ are leaves in *T*′ (and *w*_2_ and *z*_2_ are internal vertices in *T*′), edges {*w*_1_, *w*_2_} and {*w*_2_, *z*_2_} must be in *T*′ (as *w*_2_ is an internal vertex in *T*′). We remove edge {*w*_1_, *w*_2_} from *T*′ (to prevent cycles) and add edges {*z*_1_, *w*_1_}, {*w*_1_, *x*}, as well as {*w*_2_, *y*} to *T*′. This yields a valid spanning tree *T* for *G*, in which *w*_1_, *w*_2_, *z*_1_ and *z*_2_ are internal vertices, and *x* and *y* are leaves.
- *w*_1_ and *z*_2_ are leaves in *T'* (and *w*_2_ and *z*_1_ are internal vertices in *T'*). This case cannot happen because *T'* would not be connected.
- *w*_2_ and *z*_1_ are leaves in *T'* (and *w*_1_ and *z*_2_ are internal vertices in *T'*). This case cannot happen because *T'* would not be connected.
- If *w*_2_ and *z*_2_ are leaves in *T*′ (and *w*_1_ and *z*_1_ are internal vertices in *T*′), edges {*w*_1_, *w*_2_} and {*w*_1_, *z*_1_} must be in *T*′ (as *w*_1_ is an internal vertex in *T*′). We remove edge {*w*_1_, *w*_2_} from *T*′ (to prevent cycles) and add edges {*z*_2_, *w*_2_}, {*w*_2_, *y*}, as well as {*w*_1_, *x*} to *T*′. This yields a valid spanning tree *T* for *G*, in which *w*_1_, *w*_2_, *z*_1_, and *z*_2_ are internal vertices and *x* and *y* are leaves.

7. *G* has a degree-3 vertex *w* with *N*(*w*) = {*x*, *y*, *z*} such that *N*(*z*) = {*w*, *y*} and edge {*x*, *y*} ∈ *E* (as shown in Fig. 23): As *n* ≥ 7 and *G* is biconnected, there exists a vertex *u* ∈ *N*(*y*) \ {*w*, *x*, *z*} (and as *G* is biconnected, *u* lies on some path from *x* to *y*). In particular, deg(*y*) ≥ 4 in *G*. We now construct a simple and biconnected SP graph *G*′ with *n* − 1 vertices from *G* by suppressing *z* and deleting one copy of the resulting parallel edge {*w*, *y*}. We note that as deg(*y*) ≥ 4 in *G*, we have deg(*y*) ≥ 3 in *G*′. In particular, *y* is not a degree-2 vertex in *G*′, whereas *w* is. As *G*′ is a simple and biconnected SP graph with 6 ≤ *n* − 1 < *n* vertices, by the inductive hypothesis, *G*′ has a valid spanning tree *T*′, in which vertex *w* is potentially a leaf. We can now obtain a valid spanning tree *T* for *G* from *T*′ by adding the edge {*w*, *z*} to *T*′. This ensures that *w* is not a leaf in *T* (but *z* is; this is valid because *z* is a degree-2 vertex in *G*).
8. *G* has two non-adjacent degree-3 vertices *w*_1_ and *w*_2_ such that *N*(*w*_1_) = {*x*, *y*, *z*_1_}, *N*(*w*_2_) = {*x*, *y*, *z*_2_}, *N*(*z*_1_) = {*x*, *w*_1_}, and *N*(*z*_2_) = {*y*, *w*_2_} (as shown in Fig. 24): As *G* is biconnected and *n* ≥ 7, there exists a vertex *x*′ ∈ *N*(*x*) \ {*z*_1_, *w*_1_, *w*_2_, *y*}. In particular, deg(*x*) ≥ 4 in *G*. We now construct a simple and biconnected SP graph *G*′ with *n* − 1 vertices from *G* by suppressing *z*_1_ and deleting one copy of the resulting parallel edge {*x*, *w*_1_}. We note that *w*_1_ is a degree-2 vertex in *G*′, whereas deg(*x*) ≥ 3 in *G*′. By the inductive hypothesis (as *G*′ is a simple and biconnected SP graph with 6 ≤ *n* − 1 < *n* vertices), there exists a valid spanning tree *T*′ for *G*′ (potentially containing vertex *w*_1_ as a leaf). We can now obtain a valid spanning tree *T* for *G* from *T*′ by adding the edge {*w*_1_, *z*_1_}.
9. *G* has two non-adjacent degree-3 vertices *w*_1_ and *w*_2_ such that *N*(*w*_1_) = {*x*, *y*, *z*_1_}, *N*(*w*_2_) = {*x*, *y*, *z*_2_}, *N*(*z*_1_) = {*x*, *w*_1_} and *N*(*z*_2_) = {*x*, *w*_2_} (as shown in Fig. 25): In this case, we can construct a simple and biconnected SP graph *G*′ with *n* − 1 vertices from *G* by suppressing *z*_1_ and deleting one copy of the resulting parallel edge {*x*, *w*_1_} (Fig. 25). We note that *w*_1_ is then a degree-2 vertex in *G*′. By the inductive hypothesis (as *G*′ is a simple and biconnected SP graph with 6 ≤ *n* − 1 < *n* vertices), there exists a valid spanning tree *T*′ for *G*′, in which *w*_1_ is potentially a leaf (*x* cannot be a leaf in *T*′ by the inductive hypothesis, as deg(*x*) ≥ 3 in *G*′). We can now obtain a valid spanning tree *T* for *G* from *T*′ by adding the edge {*w*_1_, *z*_1_}. This ensures that *w*_1_ is not a leaf in *T*, and thus *T* is a valid spanning tree for *G*.
10. *G* has a degree-3 vertex *w* with *N*(*w*) = {*x*, *z*_1_, *z*_2_} such that there is a degree-2 vertex *y* ∈ *N*(*z*_1_) ∩ *N*(*z*_2_) and *N*(*x*) = {*z*_1_, *w*} (as shown in Fig. 26): As *G* is biconnected and *n* ≥ 7, there exists a vertex ${z}_1^{\prime}\in N\left({z}_1\right)\setminus \left\{w,x,y,{z}_2\right\}$ in *G* and ${z}_1^{\prime }$ lies on some path between *z*_1_ and *z*_2_ (as *G* is biconnected). In particular, deg(*z*_1_) ≥ 4 and deg(*z*_2_) ≥ 3 in *G*. We now construct a simple and biconnected SP graph *G*′ with *n* − 1 vertices from *G* by suppressing *x* and deleting one copy of the resulting parallel edge {*z*_1_, *w*} (Fig. 26). We note that *w* is a degree-2 vertex in *G*′, whereas deg(*z*_1_) ≥ 3 and deg(*z*_2_) ≥ 3 in *G*′. As *G*′ is a simple and biconnected SP graph with 6 ≤ *n* − 1 < *n* vertices, by the inductive hypothesis, there exists a valid spanning tree *T*′ for *G*′, in which *w* is potentially a leaf. We can now obtain a valid spanning tree *T* for *G* from *T*′ by adding the edge {*w*, *x*}, and thereby *w* becomes an internal vertex of *T* and *x* a leaf. This completes the proof.

**Lemma 9** *Let G* = (*V*, *E*) *be a simple chordal graph without cut edges and with deg*(*v*) ≥ 2 *for all v* ∈ *V. Then, for every vertex v* ∈ *V, there exist two other vertices u and w such that u*, *v and w form a triangle in G,* i.e. *such that the edges* {*u*, *v*}, {*u*, *w*} *and* {*v*, *w*} *are all in E.*

*Proof* Let *G* be a simple chordal graph without cut edges such that deg(*v*) ≥ 2 for all *v* ∈ *V*.

First, we show that every vertex belongs to a cycle. We assume that there is a vertex *v* in *V* that does not belong to any cycle. As deg(*v*) ≥ 2, *v* has at least two neighbors *a* and *b*. If we now remove the edge *e* = {*a*, *v*}, the resulting graph must still be connected; otherwise, *e* would be a cut edge, but *G* has no cut edge. However, this implies that there is a path *P* from *a* to *v* that does not use edge *e*. Therefore, re-introducing edge *e* closes a cycle. Thus, *v* belongs to a cycle in *G*.

We now assume that *v* does not belong to a triangle. Then, *v* belongs to a cycle of length at least 4. As *G* is chordal, this cycle must have a chord. Thus, *v* also belongs to a smaller cycle. Recursively, this shows that *v* must belong to a triangle, as all cycles of length larger than 3, by the definition of chordality, have a chord. This completes the proof.

## Abbreviations

GSP graph: Generalized series-parallel graph
$\mathcal{LCON}\left({N}^u\right)$: Set of leaf connecting graphs of *N*^*u*^
$\mathcal{LCUT}\left({N}^u\right)$: Leaf cut graph of *N*^*u*^
$\mathcal{LS}(G)$: Leaf shrink graph of *G*
SP graph: Series-parallel graph

## Footnotes

1 Note that for a tree, the pre-processing step would always result in a single edge.

2 See proof of Theorem 4.1 in [11].
