# Correction to: Classes of tree-based networks

**DOI:** 10.1186/s42492-021-00069-x

**Published:** 2021-01-26

**Authors:** Mareike Fischer, Lina Herbst, Michelle Galla, Yangjing Long, Kristina Wicke

**Affiliations:** 1 Institute of Mathematics and Computer Science, University of Greifswald, Greifswald, Germany; 2 School of Mathematics and Statistics, Central China Normal University, Wuhan, Hubei China

**Correction to: Vis Comput Ind Biomed Art 3, 12 (2020)**

**https://doi.org/10.1186/s42492-020-00043-z**

Following publication of the original article [1], in the description of the leaf shrinking procedure as well as in Algorithm 1 and the definition of edge-based graphs in [1], the condition that *G* contains at least two leaves (i.e., the condition |*V*_*L*_(*G*)| ≥ 2) needs to be omitted. Moreover, in line 2 of Algorithm 1 it should read $\left|V\left(\mathcal{LS}(G)\right)\right|>2$.

In particular, Algorithm 1 and Definition 3 should read as follows:

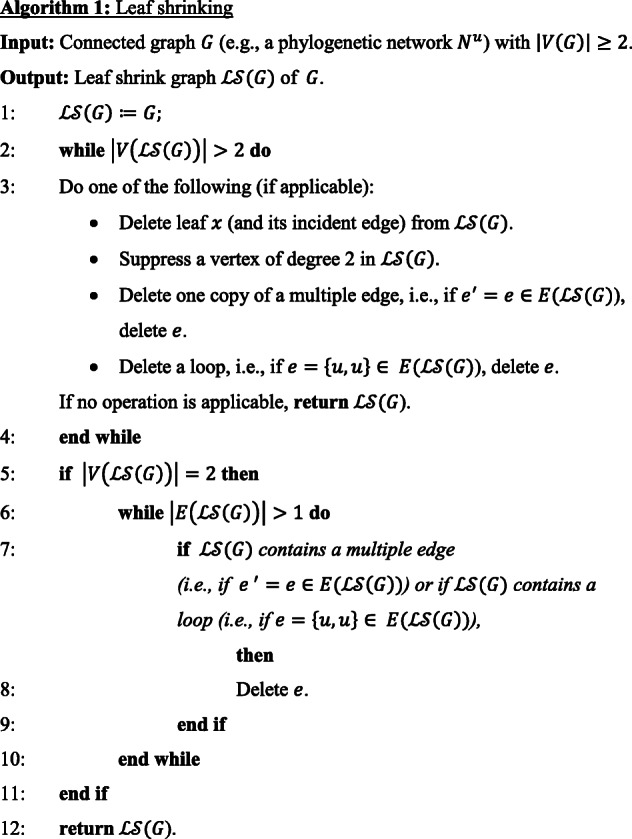



**Definition 3** Let *G* be a connected graph with |*V*(*G*)| ≥ 2. If the leaf shrink graph $\mathcal{LS}(G)$ of *G* is a single edge, *G* is called *edge-based*. Else, *G* is called *non-edge-based*. If *G* = *N*^*u*^ is a proper phylogenetic network with |*V*(*N*^*u*^)| ≥ 2 and |*X*| ≥ 2 and $\mathcal{LS}\left({N}^u\right)$ is a single edge, we call *N*^*u*^ an *edge-based* network. Else, *N*^*u*^ is called *non-edge-based*.

We thank Tom Niklas Hamann for bringing this issue to our attention.
